# Dissociating neural circuits of social and prosocial reward in rat helping behavior

**DOI:** 10.1016/j.isci.2026.114694

**Published:** 2026-01-14

**Authors:** Keren Ruzal, Estherina Trachtenberg, Ben Kantor, Hila Flumin, Adin Roemer, Andres Crespo, Johannes Kohl, Inbal Ben-Ami Bartal

**Affiliations:** 1 Sagol School of Neuroscience, Tel-Aviv University, Tel Aviv, Israel; 2 School of Psychological Sciences, Tel-Aviv University, Tel Aviv, Israel; 3 State-dependent Neural Processing Lab, The Francis Crick Institute, London, UK; 4 Nanobioengineering Group, Institute for Bioengineering of Catalonia (IBEC), Barcelona, Spain

**Keywords:** Neuroscience, Behavioral neuroscience

## Abstract

Helping behavior in rodents provides a powerful model for studying neurobiological underpinnings of prosocial motivation. Previously, rats allowed to release a trapped conspecific demonstrated prosocial motivation selectively toward ingroup members. Here, a refined version of the helping behavior test (HBT) allowed trapped rats to be freed without ensuing social contact, dissociating social from prosocial reward. In this “separated” HBT (SHBT), helping was not biased, as rats equally released ingroup and outgroup members. Whole-brain c-Fos mapping revealed a subset of the standard HBT prosocial brain network engaged in the SHBT. Observed absence of activity in the nucleus accumbens (NAc) instigated a chemogenetic investigation of this region. NAc activity was not necessary for helping, but significantly reduced affiliative behavior. The recruitment of *Oxtr+* cells in sensory cortices suggests modulated sensory processing. Together, these findings dissociate neural circuits underlying social and prosocial motivation and provide a mechanistic framework for studying mammalian prosocial behavior.

## Highlights

• Rats similarly release ingroup and outgroup conspecifics without social reward
• Helping without social reward engages the prosocial brain network but not the NAc
• NAc inhibition reduces affiliative behavior but not helping behavior
• Helping recruit *Oxtr+* cells in sensory but not frontal cortices

## Graphical abstract

**Figure:**
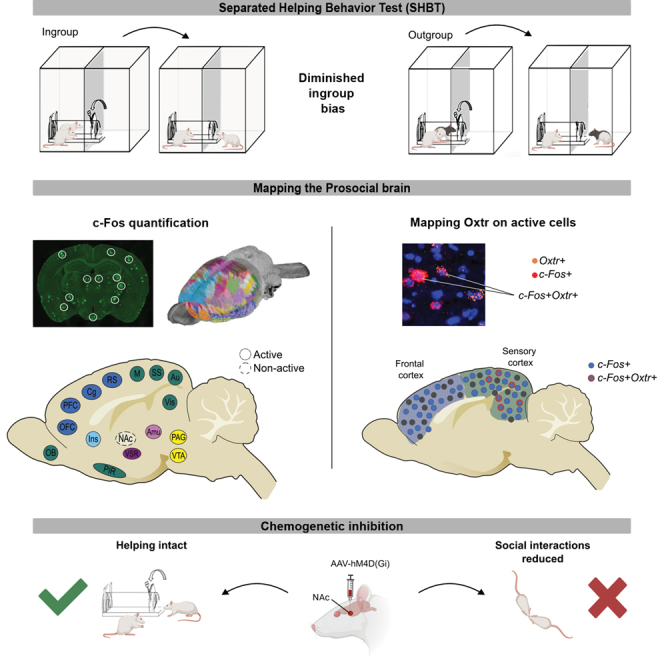

## Introduction

The survival and thriving of social species rely on maintained prosocial behavior between conspecifics. A prosocial response to others in need or distress enables genetic continuity^1^^,^^2^^,^^3^ and has evolved in altricial species in the context of parental care as well as the broader social group. Empathy, which involves recognizing and sharing others’ affective state, can be a powerful driver of prosocial behavior when coupled with a motivation to improve others’ well-being.^4^^,^^5^ Similarities in the neurobiological mechanisms underlying empathy across species^6^^,^^7^^,^^8^^,^^9^^,^^10^^,^^11^^,^^12^ indicate that engaging affective and motivational circuits is an effective proximate mechanism to promote prosocial acts.^13^^,^^14^^,^^15^^,^^16^

Prosocial acts can arise from diverse motivational drivers, including tension reduction, social reward received from the helped individual, and intrinsic prosocial reward, the “warm glow” associated with helping itself. These distinct motivational states rely on partially overlapping but separable neural processes, together forming the complex mechanism that transforms observed distress into helping behavior. Importantly, distinguishing between social motivation (the drive to seek affiliative contact) and prosocial motivation (the drive to alleviate another’s distress) is important for developing a conceptual framework for understanding the underlying neural circuitry.

The rat helping behavior test (HBT) is an ideal paradigm for investigating this process. During the HBT, rats can release a trapped conspecific by opening a restrainer door.^17^ Following door-opening, the two rats remain in the arena together for the rest of the session. Brain-wide quantification of the immediate-early-gene c-Fos has revealed that, during the HBT, “helpers” activate a distinct neural network in the presence of a trapped cagemate,^7^ which is strikingly similar to that described in human empathy.^5^ This “prosocial brain network” includes the anterior cingulate and insular cortices (ACC and AI, respectively), as well as frontal and limbic regions and parts of the reward system, including the Nucleus accumbens (NAc).^18^^,^^19^

In this study, we sought to dissociate the neural activity involved in the expectation of social reward from interaction with the released rat from the activity associated with the outcome of the conspecific. To this end, a modified version of the HBT was used, where a divider separates the two rats after door-opening (“separated HBT” or SHBT). Previous observations from this^17^ and other paradigms^20^^,^^21^ have demonstrated that social reward is not necessary for helping, with rats persisting to release conspecifics even when post-release contact is not afforded. As rats in the SHBT do not expect to be united after door-opening, their neural activity will reflect the prosocial response to the conspecific’s distress, dissociated from the expectation of social reward.

To further our understanding of the neural signature of prosocial motivation, we quantified c-Fos across the brain using an in-house software,^22^ and examined the distribution of oxytocin receptors (*Oxtr*) in conjunction with neural activity using multiplex RNAscope. This neuromodulatory system holds particular importance for prosocial behavior.^23^^,^^24^ The neuropeptide oxytocin (OT), secreted from supraoptic, paraventricular, and accessory nuclei of the hypothalamus, is the main neuromodulator associated with social behavior, which was discovered in the context of birth, lactation, and maternal care.^25^^,^^26^^,^^27^^,^^28^ OT plays a key role in prosocial behaviors such as bonding, attachment, and mating,^26^^,^^29^ promotes a “safety” mode in social contexts,^30^^,^^31^^,^^32^ and affects social salience and reward processing.^33^ To identify which parts of the “prosocial brain network” are sensitive to OT signaling, we mapped *Oxtr* distribution within active neurons.

We found that across strain and sex, helping behavior occurred in the SHBT for about half the rats, in line with previous evidence showing helping is not primarily motivated by social reward. In contrast with the HBT, where rats seldom help outgroup members,^18^^,^^19^^,^^34^ a similar ratio of helpers was observed independently of the social identity of the trapped rat in the SHBT, with rats helping strangers of an unfamiliar strain as well as cagemates. A quantification of brain-wide c-Fos expression revealed that the “prosocial brain network” was active during the SHBT, including sensory regions, the AI and ACC, and frontal regions. However, the NAc, a central hub of the reward system and typically engaged in the HBT, was not more active than baseline during the SHBT.

To investigate this surprising finding, we conducted a series of chemogenetic manipulations of the NAc and found that helping was not abolished by NAc inhibition. Nevertheless, NAc inhibition significantly reduced affiliative behavior, and c-Fos levels in the NAc shell (NAc-sh) positively correlated with helping behavior in the task, suggesting it generally represents social reward, and includes a sparse population that predicts prosocial behavior. Finally, examining co-labeled *c-Fos+/Oxtr+* cells, we found that although c-Fos was recruited in both sensory and frontal regions, only sensory cortices recruited oxytocin-sensitive subpopulations during the SHBT.

In summary, this work highlights the neural activity involved in prosocial behavior in rats. These data reveal that helping without the expectation of social interaction occurs toward ingroup and outgroup members, involves activity in a subset of regions of the prosocial brain network, and recruits oxytocin-sensitive subpopulations in sensory regions.

## Results

### Rats similarly release trapped ingroup and outgroup conspecifics in the separated helping behavior test

**Figure 1:**
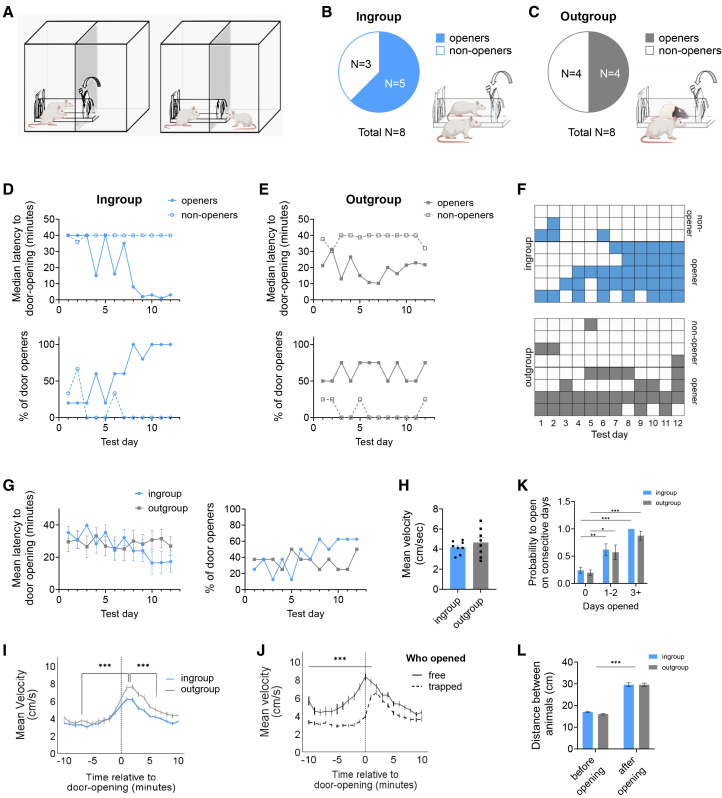
Rats similarly release a trapped ingroup or outgroup conspecific in the separated helping test (A) A diagram of the separated helping behavior test (SHBT). After door-opening, rats remain in separate spaces, minimizing post-release contact. (B and C) Opening proportions in ingroup and outgroup conditions. About half of the rats became openers in both conditions. (D and E) The pattern of the door opening for openers and non-openers over test days is shown for ingroup and outgroup conditions, demonstrating similar trends in both social conditions. (F) Door-openings are shown per rat. Clear squares represent days without door-openings, colored squares represent days with door-openings. (G) The overall opening latency was similar in both social conditions. (H) Activity levels before door-opening were similar in both social conditions. (I) Activity levels increased around door-opening in both ingroup and outgroup conditions, demonstrating that freeing the trapped rat was a salient event despite the lack of social contact in the SHBT. (J) Activity levels of the free rat were increased even when it wasn’t the one opening the door, pointing to the saliency of the event. (K) The probability of rats opening on a given day increased the more they had previously opened, demonstrating that opening was reinforcing for both ingroup (blue) and outgroup (gray) conditions. (L) The distance between free and trapped rats increased after door-opening, demonstrating that opening was not motivated by a desire for social contact. ∗*p* < 0.05 ∗∗*p* < 0.01 ∗∗∗*p* < 0.001. All error bars represent SEM. Statistical analyses included Fisher’s exact tests for opening proportions, Friedman tests for repeated measures of door-opening latency, Cochran’s Q tests for repeated binary opening outcomes, two-way repeated-measures ANOVA with Bonferroni-corrected post hoc comparisons for effects of social condition and test day, unpaired two-tailed *t*-tests for activity measures, and mixed-model ANOVA for time-resolved activity analyses. Illustrations were made using BioRender.

To determine the effects of social identity on prosocial motivation in the absence of post-release interaction, Sprague Dawley (SD) adult male rats were tested in the SHBT with a trapped conspecific who was either a male SD cagemate (“ingroup,” *n* = 8) or a male stranger of the unfamiliar Long-Evans (LE) strain (“outgroup,” *n* = 8). An additional cohort was tested without any trapped rat in the restrainer (“empty,” *n* = 8). During 12 daily 1-h sessions, rats could release the trapped conspecific by pulling the restrainer door from the side. This allowed the trapped rat to escape into a part of the arena that was separated by a clear perforated divider (Figure 1A and Video S1). In order to prevent learned helplessness, if the rat did not open the restrainer after 40 min, the door was opened halfway by the experimenter, allowing the trapped rat to easily escape. Door-opening was quantified prior to this time point. We found that, similarly to the original HBT, most rats were motivated to release a trapped cagemate, and became “openers,” releasing the trapped rat on at least a third of testing sessions in the ingroup condition (*n* = 5/8 openers, 62.5%, Figure 1B). Interestingly, the outgroup condition had a similar opening ratio (*n* = 4/8 openers, 50%, Figure 1C; Fisher’s exact test, ingroup vs. outgroup, *p* = 1.00). None of the rats tested in the “empty” condition learned how to open the door. Open-field behavior measured prior to SHBT testing was similar across openers and non-openers, indicating that trait anxiety does not explain differences in helping behavior (Figures S1A–S1C). Similarly, boldness scores (time to approach a lid of a half-opened cage, see Methods) measured prior to the SHBT suggest that dominance does not explain differences in helping behavior (Figures S1D–S1F). By the final testing sessions, ingroup openers were releasing the trapped cagemate consistently and quickly, as demonstrated by a significant decrease in door-opening latency (Friedman’s χ^2^(11) = 29.68, *p* = 0.002) and increased percent of door-openings (Cochran’s Q(11) = 27.68, *p* = 0.004; Figure 1D). In the outgroup condition, the latency to open and percent openings did not significantly change across testing days (χ^2^(11) = 9.08, *p* = 0.61; Q(11) = 4.21, *p* = 0.96; Figure 1E), reflecting less consistent helping within rats (Figure 1F). Nevertheless, mean opening patterns were similar for the ingroup and outgroup condition (Two-way RM ANOVA, Social condition F(1,14) = 0.09, *p* = 0.76; test day F(11,154) = 1.32, *p* = 0.22; interaction F(11,154) = 1.89, *p* = 0.04; post-hoc Bonferroni’s multiple comparisons test, *p* > 0.938 for all comparisons; Figure 1G), and activity levels were similar in both conditions (t(14) = 0.88, *p* = 0.39; Figure 1H).

Video S1. Rats tested in the SHBT, related to Figure 1

### Releasing the trapped rat is reinforcing for the free rat

We examined the rats’ movement patterns to determine the motivational state of the free rat. We found that activity levels were significantly elevated in the minutes after door-opening in both social conditions (MMA, Ingroup, F(20,1950.28) = 4.07, *p* < 0.001, Outgroup, F(20,1942.31) = 7.88, *p* < 0.001, Figure 1I), indicating that this was a salient event. Moreover, in the minutes before door-opening, activity was significantly higher when the free rat opened the restrainer compared to self-release by the trapped rat occurring after the halfway door-opening (MMA, F(20,1974.32) = 7.33, *p* < 0.001; Figure 1J), suggesting that the free rats’ activity was specifically targeted at door-opening. Furthermore, the probability for door-opening increased the more rats had previously opened the restrainer, further evidencing the reinforcing value associated with releasing the trapped rat (ANOVA, F(2,175) = 42.54, *p* < 0.001; Figure 1K). This effect was similar for both social conditions (F(1,175) = 1.01, *p* = 0.32; Figure 1K), and occurred even though the distance between rats significantly increased following door-opening (two-way ANOVA, F(1,14) = 503.4, *p* < 0.001; Figure 1L). These combined results demonstrate that even though rats were separated after door-opening, releasing the trapped rat was reinforcing regardless of the social condition.

### Brain-wide neural activity associated with the separated helping behavior test is similar across social conditions

**Figure 2:**
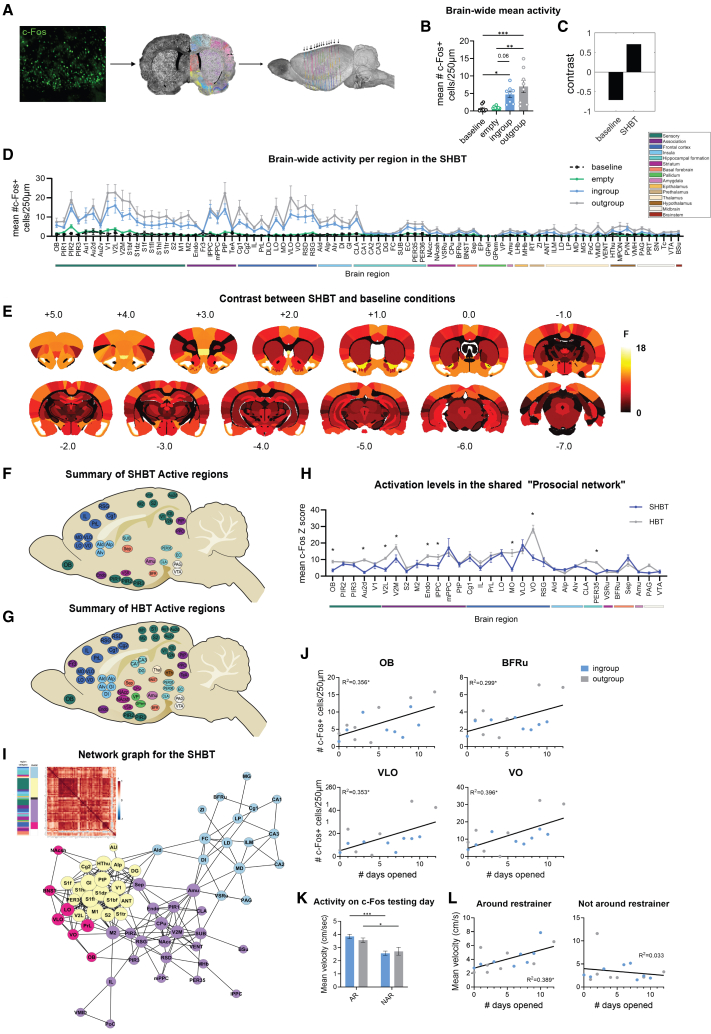
A similar neural activity pattern was observed in both ingroup and outgroup conditions in the SHBT (A) A diagram of the quantification process using the “Brainways” software. (B) Mean brain-wide activation was observed in ingroup and outgroup conditions compared to baseline and empty conditions. (C) PLS analysis revealed a significant LV for SHBT compared to baseline; bars display the contrast. (D) Analysis per region showed that mainly sensory, frontal, and isular regions were activated in ingroup and outgroup SHBT conditions compared to baseline and empty conditions. (E) Brain-wide contrast of neural activity in the SHBT compared to baseline. (F) Summarizing diagrams of the brain regions significantly activated in the SHBT, (G) and in the HBT, compared to baseline. (H) Differences in activation levels within the shared network of the HBT and the SHBT. To control for staining differences across labs, activation levels were normalized to baseline c-Fos from undisturbed rats in each dataset. (I) Network graph depicts the top 10% inter-region correlations for the SHBT, based on Louvain clustered heatmap of pairwise correlation values for the SHBT (a higher resolution image can be found in Figure S3C). The bars on the left visualize the identified clusters and regions’ categories. Circle color represents clusters identified via the Louvain algorithm, circle size represents the number of degrees for each region. (J) A subset of regions was positively correlated with helping behavior in the SHBT. (K) Velocity on the day of c-Fos data acquisition was higher when rats were around the restrainer in both social conditions (L) and was correlated with helping behavior throughout the test. ∗*p* < 0.05 ∗∗*p* < 0.01 ∗∗∗*p* < 0.001. All error bars represent SEM. Statistical analyses included one-way ANOVA with Tukey’s multiple comparisons tests for brain-wide activation, partial least squares (PLS) analysis with permutation testing for latent variables, unpaired two-tailed t-tests with FDR correction for region-by-region activation, Pearson’s correlation coefficients for associations between neural activity and behavior, and one-way ANOVA for velocity comparisons.

To outline the brain-wide neural activity associated with the SHBT, brains from rats tested in the experiment described above were collected immediately after the final testing session and immunostained for c-Fos, as an index of neural activity (Figure 2A). Using our in-house software for the automated registration and quantification of fluorescence on coronal slices,^22^ c-Fos levels were compared between ingroup (*n* = 8), outgroup (*n* = 8), empty restrainer (*n* = 8), and a baseline condition of naive, age- and weight-matched SD male rats (*n* = 8). In an overall analysis of all sampled areas, mean brain c-Fos levels were significantly elevated in the social conditions compared to baseline and empty conditions (ANOVA; F(3, 28) = 9.128, *p* < 0.001; Tukey’s multiple comparisons test, baseline vs. ingroup, *p* = 0.04; baseline vs. outgroup, *p* < 0.001; ingroup vs. empty, 0.06; outgroup vs. empty, *p* = 0.001; Figure 2B). No difference was observed between the social conditions (*p* = 0.39) or between baseline and empty conditions (*p* = 0.99). Next, to determine whether a distinct neural pattern was associated with task conditions, a partial least squares (PLS) task analysis^35^^,^^36^ was conducted. A significant latent variable was revealed for the contrast between the SHBT compared to baseline conditions (LV1 *p* < 0.001; Figure 2C), but not when contrasting ingroup and outgroup conditions (*p* = 0.24), indicating that a similar pattern of activity was observed across ROIs for the SHBT conditions (Figure 2D; see Figure S2 for c-Fos data per region; Table S1 for regions' abbreviations). Similarly, there was no brain-wide effect for opening status, suggesting it does not explain variance in brain-wide activity in the HBT (Figure S3B). Thus, in line with the similar rates of door-opening described above, brain-wide neural activity in the SHBT was not significantly modulated by the social condition, and data from the ingroup and outgroup conditions were pooled for the rest of the analyses.

### Prosocial network associated with the separated helping behavior test

Region-by-region comparison of neural activity in the SHBT and baseline conditions revealed significant activation in a dispersed network including sensory cortices (olfactory, auditory, visual, somatosensory, and motor regions), associative, frontal, and insular cortices (*t* test with FDR correction; significance threshold *p* < 0.006; Figures 2E, 2F and Table S2). To identify regions differentiating social and prosocial reward, we compared previously published c-Fos data from SD male rats in the ingroup HBT^7^ with our data from the ingroup SHBT. The HBT activated a broader network than the SHBT (Figures 2F and 2G), and several regions within the shared network (OB, Au2d, V2L, V2M, Endo, lPPC, MO, VO, and PER35) were significantly more activated in the HBT (Figure 2H). This suggests that certain regions are more involved in social rather than prosocial motivation.

To assess functional connectivity for the SHBT condition, network graph analysis was performed based on inter-region Pearson’s correlations (Figures 2I and S3C). Louvain community detection analysis revealed four clusters: one was mainly composed of sensory regions, another was largely thalamic, and two clusters consisted of a mix of frontal and limbic areas. Based on centrality parameters, four regions emerged as central hubs: the Amygdala (Amu), Secondary motor area (M2), Posterior area of the agranular insular cortex (AIp), and Hypothalamus (HThu), having the top 20% combined score for degree and betweenness centrality (see the STAR Methods section for details of the network analysis).

### Neural activity associated with helping behavior in the separated helping behavior test

To determine which brain regions were specifically associated with helping behavior, the significantly active ROIs identified above were examined for correlation with door-opening during the SHBT. c-Fos levels in several regions significantly positively correlated with door-opening, including the olfactory bulbs (OB), ventral and ventrolateral parts of the orbitofrontal cortex (VO, VLO), and the Basal forebrain (BFRu) (Figure 2I). These correlations were maintained after outlier analysis. These areas are thus associated with prosocial motivation and provide targets for further experiments that will be needed to establish whether this activity is causally involved in helping.

An alternative explanation for the association between brain activity and behavior is that c-Fos levels may reflect motor activity rather than prosocial motivation. Yet motor activity can also emerge due to prosocial motivation, if directed at releasing the trapped rat. Indeed, examining rats’ activity levels, we observed that velocity was significantly higher when rats were in the area around the trapped rat compared to when they were outside this zone (ANOVA, F(1,959) = 29.08, *p* < 0.001; Figure 2J). This indicates that activity was specifically targeted at the trapped rat and motivated by its presence. Furthermore, activity around the restrainer was positively correlated with the number of door-openings (Pearson’s r = 0.62, *p* = 0.01; Figure 2K), but not with activity outside this area (r = −0.18, *p* = 0.52; Figure 2K). This suggests that conspecific-focused activity was prosocial in nature.

### Brain-wide mRNA analysis of c-Fos and oxytocin receptors co-expression in the separated helping behavior test

**Figure 3:**
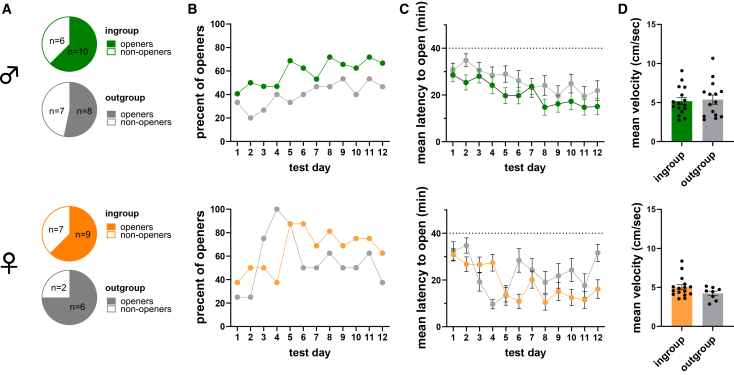
Similar levels of helping behavior in male and female LE rats were tested in the SHBT (A) Similar opening proportions in ingroup and outgroup conditions in male (top) and female (bottom) LE rats. (B) Similar door-opening percentages for rats tested in the ingroup (green/orange) and outgroup (gray) conditions in males (top) and females (bottom). (C) Similar door-opening latencies for ingroup and outgroup conditions in both sexes. The dashed line represents the end of the session at 40 min. (D) Similar velocity of free rats before door-opening between social conditions in both sexes. All error bars represent SEM. Statistical analyses included Fisher’s exact tests for opening proportions, Cochran’s Q tests for repeated measures of opening probability, Friedman tests for repeated measures of door-opening latency, and unpaired two-tailed t-tests for velocity comparisons.

We next sought to investigate the involvement of the oxytocinergic system during the SHBT. To that end, we conducted a brain-wide multiplex RNAscope analysis to co-label oxytocin receptor (*Oxtr*) and *c-Fos* expression in brains from another cohort of rats tested in the SHBT. Here, adult male and female LE rats were tested with either a trapped conspecific of the same sex who was either an LE cagemate (“ingroup”; *n* = 16 males, 16 females) or an SD stranger (“outgroup” *n* = 16 males, 8 females). As in the findings reported above, in this cohort around half the rats became openers (*n* = 31/56 openers, 55%), and no effect of sex was observed (Fisher’s exact test *p* = 0.78; males, *p* = 0.72; females, *p* = 0.21; Figure 3A). Open-field tests conducted prior to testing in the SHBT did not find differences between openers and non-openers in either of the sexes, indicating similar baseline anxiety levels across these phenotypes (Figures S1G–S1L). Similarly, boldness scores measured as an index for dominance prior to the SHBT found no difference in boldness between openers and non-openers, indicating that dominance does not explain differences in helping behavior in either of the sexes (Figures S1M–S1R).

Reproducing the previous experiment, opening patterns were similar across social conditions, with similar opening ratios (Fisher’s exact test, *p* = 0.59, Figure 3A), percent openings (male ingroup, Q(11) = 26.34, *p* = 0.06; male outgroup, Q(11) = 14.3, *p* = 0.22; female ingroup, Q(11) = 31.47, *p* < 0.001; female outgroup, Q(11) = 22.82, *p* = 0.02; Figure 3B), and opening latencies (male ingroup, χ^2^(11) = 30.8, *p* = 0.01, male outgroup, χ^2^(11) = 31.97, *p* < 0.001, female ingroup, χ^2^(11) = 50.71, *p* < 0.001, female outgroup, χ^2^(11) = 23.8, *p* = 0.01, Figure 3C). Additionally, no differences were observed in activity levels between social conditions (males, t(1,13) = 0.89, *p* = 0.39; females, t(1,22) = 1.91, *p* = 0.069; Figure 3D). These results provide further validation that rats tested in the SHBT are motivated to release a trapped conspecific, regardless of social identity.

**Figure 4:**
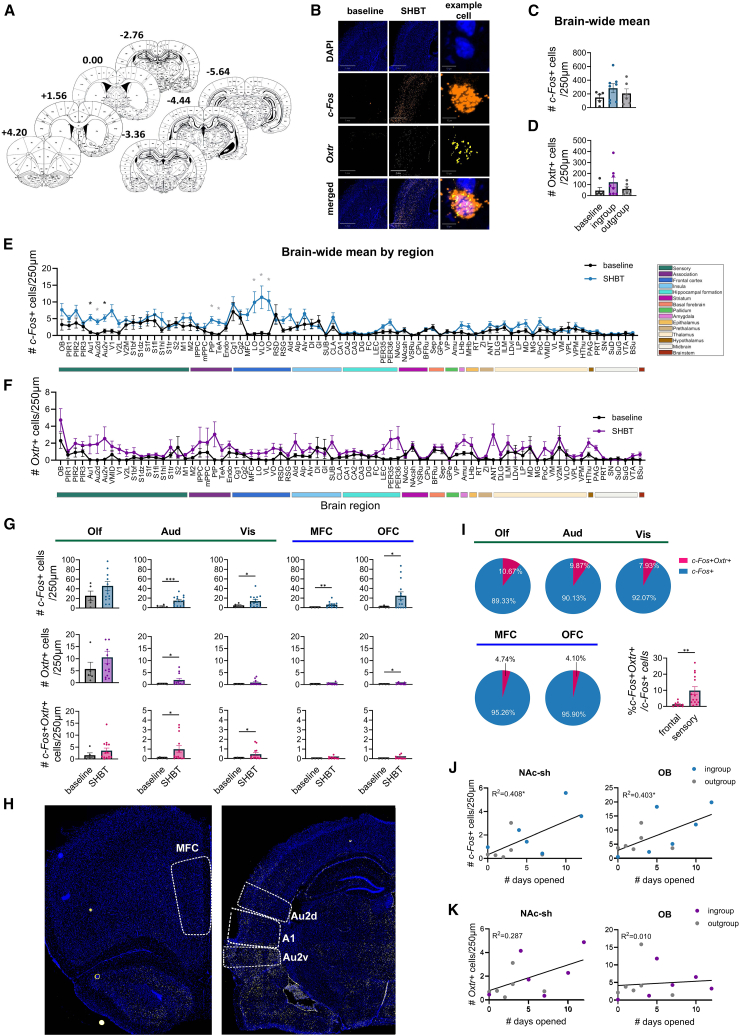
Increased *c-Fos* and *Oxtr* transcription levels in the SHBT (A) Coordinates of sampled tissue used for the RNAscope quantification of *c-Fos* and *Oxtr*. (B) Representative images of RNAscope staining. Scale of left and middle columns: 1 mm. Scale of right column: 10 μm. (C) No differences were observed in brain-wide *c-Fos* or (D) *Oxtr*+ cells. (E) Per-region mixed model analysis revealed a subset of regions significantly activated in the SHBT, (F) but none of the regions reached significance in general *Oxtr+* cell counts. Black asterisks represent significant differences between baseline and SHBT after FDR correction; gray asterisks represent significance before FDR correction. See Table S3 for full statistics. (G) Within the activated network, only auditory and visual regions showed higher numbers of *c-Fos+Oxtr+* cells compared to baseline. Olf, composed of OB, Pir1, Pir2, Pir3 and Endo; Aud, composed of Au1, Au2d, Au2v; Vis, composed of V1, V2L, V2M; OFC, composed of VO, LO, VLO; MFC, composed of PrL, IL. (H) Representative images of *Oxtr+* staining in the medial frontal cortex (MFC; top) and auditory cortex (A1, Au2d, Au2v; bottom). (I) Percentages of *c-Fos+Oxtr+* cells were higher in sensory regions than in frontal regions. (J) The Nucleus accumbens shell (NAc-sh) and olfactory bulbs (OB) were the only regions whose activation was positively correlated with helping in the SHBT. (K) Only the NAc-sh also showed a trend of correlation between *Oxtr+* cell numbers and helping. ∗*p* < 0.05 ∗∗*p* < 0.01 ∗∗∗*p* < 0.001. All error bars represent SEM. Statistical analyses included one-way ANOVA for brain-wide expression levels, unpaired two-tailed t-tests with false discovery rate (FDR) correction for region-by-region analyses, Welch-corrected t-tests where variances were unequal, and Pearson’s correlation coefficients (with Spearman’s correlations reported for robustness) for associations between gene expression and helping behavior. Black asterisks indicate significance after FDR correction, and gray asterisks indicate significance prior to correction.

After the final SHBT session, brains from a subset of rats were analyzed via RNAscope staining for *c-Fos* and *Oxtr* expression (*n* = 5 “baseline”; *n* = 8 “ingroup”; *n* = 6 “outgroup,” half male and half female in each group, Figures 4A and 4B). Analysis of mean brain-wide *c-Fos* and *Oxtr* expression levels found no main effect for condition for either marker (*c-Fos*, F(2,16) = 1.14, *p* = 0.34, Figure 4C; *Oxtr*, F(2,16) = 1.16, *p* = 0.34, Figure 4D). Thus, as in the previous experiment, data from the ingroup and outgroup were pooled for the following analyses.

### Analysis of *c-Fos and* oxytocin receptors mRNA in the separated helping behavior test compared to baseline

When examining expression levels per brain region, most regions did not show an effect in SHBT compared to baseline. However, significantly higher *c-Fos* levels were observed in the auditory cortex (*t* test with FDR correction; significance threshold *p* < 0.00025; Figure 4E and Table S3). The Orbitofrontal cortex and some associative regions also displayed strong activation (PtP, TeA, Au2v, VO, VLO, LO); however, they did not retain significant activation following the FDR correction. When examining *Oxtr* expression levels per brain region, no significant differences emerged between SHBT and baseline conditions (*t* test with FDR correction; Figure 4F and Table S4).

### Sensory, but not frontal, oxytocin receptors*+* subpopulations are activated in the separated helping behavior test

We next investigated *Oxtr* expression in the ROIs identified above (sensory and frontal cortices). To this end, we quantified *c-Fos*, *Oxtr* and *c-Fos+Oxtr+* co-labeled cells specifically in Olfactory (Olf; composed of OB, Pir1, Pir2, Pir3 and Endo; See Table S1 for regions’ abbreviations), auditory (Aud; composed of Au1, Au2d, Au2v), and visual (Vis; composed of V1, V2L, V2M) cortices, as well as Orbitofrontal cortex (OFC; composed of VO, LO, VLO) and medial frontal cortex (MFC; composed of PrL, IL). This analysis revealed that *c-Fos* levels were significantly higher for all these “parent” regions (Vis, t(16.73) = 2.43, *p* = 0.027; Aud, t(16.33) = 4.096, *p* < 0.001; MFC, t(11) = 3.55, *p* = 0.005; OFC, t(13.36) = 2.92, *p* = 0.012; Figure 4G), barring the Olf which did not reach significance (t(17) = 1.22, *p* = 0.24). *Oxtr+* cells constituted a smaller population than *c-Fos+* cells, and significantly increased levels were found in Aud and OFC (t(11.18) = 2.31, *p* = 0.041; t(10.62) = 2.46, *p* = 0.032, respectively; Figure 4G).

A significantly higher number of *c-Fos+Oxtr+* co-labeled cells were found in the Auditory (Welch-corrected t(12.18) = 2.46, *p* = 0.03) and Visual cortices (Welch-corrected t(11.37) = 2.21, *p* = 0.048). In contrast, the MFC and OFC showed no such increase (t(12) = 1, *p* = 0.33; t(10) = 1.98, *p* = 0.076, respectively; Figures 4G and 4H). Furthermore, the percentage of *c-Fos+Oxtr+* cells was higher in the sensory cortices compared to the frontal cortices (Welch-corrected t(14.01) = 3.41, *p* = 0.004; Figure 4I). These findings suggest that during the SHBT, a specific sub-population of *Oxtr+* cells was recruited in the sensory cortex, but not in the frontal cortex.

Finally, to determine whether *c-Fos* or *Oxtr* levels were associated with helping in the SHBT, we examined correlations between door-openings and these indices for each brain region. A positive correlation was identified between *c-Fos* and the number of door openings in two regions: the OB and the NAc-sh. This correlation was significant according to Pearson’s test (NAc-sh, r = 0.64, *p* = 0.025; OB, r = 0.63, *p* = 0.027) and showed a strong trend according to Spearman’s test (NAc-sh, r = 0.52, *p* = 0.08; OB, r = 0.57, *p* = 0.06; Figure 4J). Associations were approximately linear with homoscedastic residuals and no influential points; Pearson’s r is therefore the appropriate primary test. Spearman’s ρ is provided for robustness. For *Oxtr,* a trend for a positive correlation with door-openings was observed for the NAc-sh and no other region (Pearson’s test, NAc-sh, r = 0.54, *p* = 0.07; OB, r = 0.10, *p* = 0.76; Spearman’s test, NAc-sh, r = 0.49, *p* = 0.11; OB, r = 0.35, *p* = 0.27; Figure 4K). Thus, these regions should be investigated further for a role in prosocial motivation.

### Nucleus accumbens inhibition reduced affiliative behavior but not door-opening

The NAc was previously identified as a central hub in the “prosocial brain network” activated during the HBT.^7^^,^^18^^,^^19^ Surprisingly, in the SHBT, this region did not show significantly elevated activity compared to baseline. To causally investigate the role of the NAc in helping behavior, NAc activity was manipulated chemogenetically in adult male LE rats tested with a trapped ingroup member (an unfamiliar rat of the same strain) in the original HBT, where rats are not separated by a divider post-release.

### Manipulating nucleus accumbens activity in expert openers

**Figure 5:**
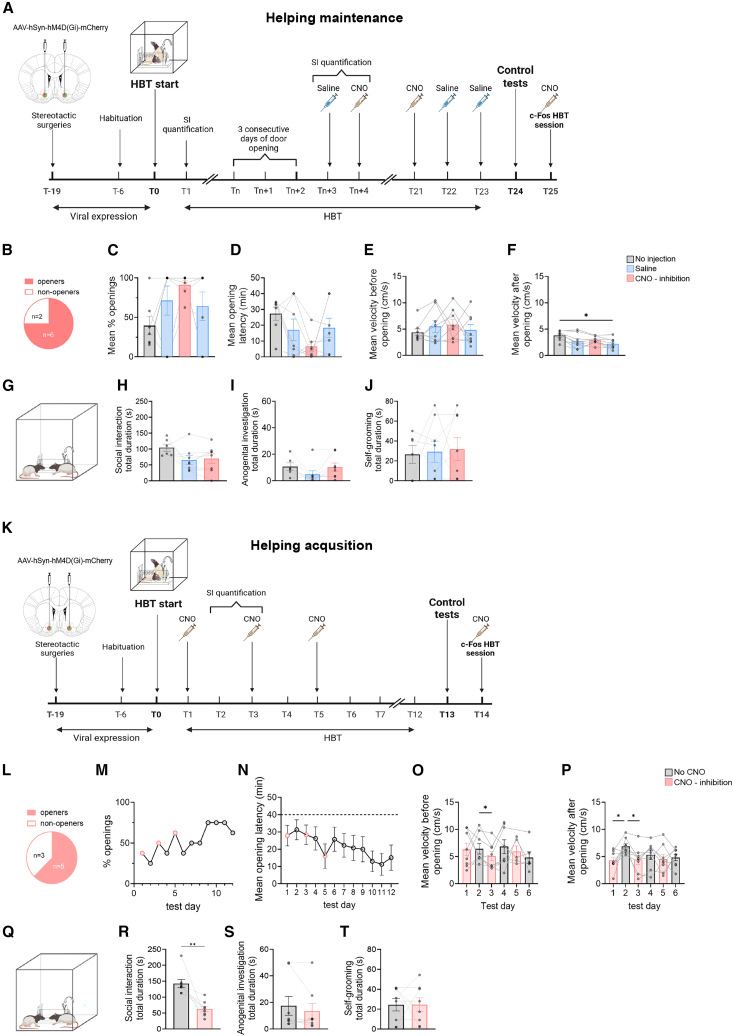
Chemogenetic manipulations did not disrupt door-opening, yet significantly reduced affiliative behavior (A) Timeline of experiment 1: NAc chemogenetic inhibition in “expert” door-openers (after 3 consecutive openings) tested in the original (non-separated) HBT. Average day to reach criterion: 11.5 ± 5.13. After all rats received at least 3 days of CNO injection, all rats were tested in another two sessions of Saline injections. (B–F) No significant effect of the inhibition on behavior in the HBT: percentages of door-opening increased (B and C), and average door-opening latencies (D). CNO did not alter velocity levels before (E) or after (F) door-opening. (G–J) Quantification of anogenital sniffing, general interaction, and self-grooming in the first 10 min after door-opening on no injection, saline, and CNO sessions reveals no effect for NAc inhibition during the late stages of the HBT. (K) Timeline of experiment 2: chemogenetic NAc inhibition in “non-expert” door-openers (during the acquisition of the behavior in the first sessions of the HBT). (L–P) No effect of the inhibition on behavior in the HBT. (Q–T) Quantification of anogenital sniffing, general interaction, and self-grooming during the first 10 min after door-opening on saline and CNO reveals an effect on social interactions when inhibiting the NAc during the early stages of the HBT. ∗*p* < 0.05 ∗∗*p* < 0.01. All error bars represent SEM. Statistical analyses included repeated-measures ANOVA for within-rat comparisons across injection conditions, Friedman tests for repeated measures of door-opening latency, paired or unpaired two-tailed t-tests for behavioral comparisons as appropriate, and post hoc multiple-comparisons corrections where applicable. Illustrations were made using BioRender.

First, we examined whether NAc manipulation was required for maintaining door-opening in established openers. Bilateral injections of inhibitory designer receptors activated only by designer drugs (DREADDs) were performed in the NAc (A/P: 1.5, M/L: ±1.5, D/V: −8.0; *n* = 8, AAV8-hSyn-hM4D(Gi)-mCherry) three weeks prior to behavioral testing (Figure 5A). The ratio of c-Fos+ neurons was successfully modulated by 3 mg/kg Clozapine-N-Oxide (CNO) i.p. administration (Figures S5A and S5B; summary of viral spread Figure S4A), demonstrating the effectiveness of the manipulation. A rat was considered an “opener” after three consecutive door-opening days. The range of days to reach the criterion varied considerably between individual rats (1–17 days, mean = 11.5, SD = 5.13), and the total length of the HBT lasted 23 days (see Figure S5C for timeline per rat). One rat in the inhibition condition never opened the restrainer and was excluded. After reaching the criterion, rats were alternately treated with saline or CNO over the next testing sessions (Figure 5A). Saline trials served to exclude behavioral changes due to the injection itself. We found that CNO administration did not significantly reduce door-opening, as expressed by the number of openers, door-opening ratio, and latency before, during, and after CNO administration (Figures 5B–5D). Similarly, no significant differences were observed in activity levels prior to door-opening (Figure 5E). A significant decrease in activity levels was observed after door-opening (ANOVA, F(3,28) = 3.11, *p* = 0.04), but this effect was not driven by CNO administration, as no difference was observed between CNO and saline sessions (Figure 5F).

Another cohort of rats was injected with an excitatory DREADD to exclude the option of opening facilitation (*n* = 8, AAV8-hSyn-hM3D(Gq)-mCherry, see Figure S4B for viral spread). However, no effects on helping behavior or activity levels were observed (Figures S5D and S5E).

To examine whether NAc inhibition may have altered social reward, affiliative social interaction (sniffing and following; SI), anogenital investigation, and self-grooming were quantified during the first 10 min after opening in three sessions of the HBT. We compared three conditions: a “no injection” session, a “saline injection” session, and a “CNO injection” session (Figure 5A). A within-rat comparison revealed no differences in any of the measures (social interaction, F(1.37, 8.19) = 2.83, *p* = 0.13; anogenital investigation, F(1.66, 9.96) = 2.92, *p* = 0.11; self-grooming, F (1.88, 17.85) = 0.056, *p* = 0.94; Figures 5G–5J), suggesting that NAc inhibition in the later stages of the HBT does not alter post-opening social interactions.

Finally, CNO administration did not affect motor activity, anxiety, or non-social reward-seeking or consumption in tests performed after the HBT (Figures S5F and S5G). These results indicate that NAc supports affiliative social behavior, but it is not necessary for door-opening to occur.

### Manipulating nucleus accumbens during the acquisition of door-opening

As NAc manipulation did not disrupt helping in expert openers, we sought to determine whether NAc activity was required for the initial acquisition of door-opening. To this end, another cohort of adult male LE rats (*n* = 8) was tested in the HBT under the chemogenetic inhibition of NAc activity (AAV8-hSyn-hM4D(Gi)-mCherry, Figure 5K; see Figure S4C for viral spread). HBT testing began three weeks after the virus injection, and CNO was administered in alternating sessions (test days 1, 3, 5), and on the final session for histological validation (Figure 5K). Most rats (*n* = 5/8, 62.5%) became openers (Figures 5L and 5M), and door-opening latency was significantly reduced across testing sessions (χ^2^(11) = 28.38, *p* = 0.003, Figure 5N). There was no change in activity levels following CNO administration, neither before nor after door-opening on the first week of testing (Figures 5O and 5P). Thus, NAc inhibition from the task onset did not impede door-opening in the HBT.

To examine whether NAc inhibition may have altered social reward, affiliative social interaction (sniffing and following; SI), anogenital investigation, and self-grooming were quantified during the first 10 min after opening in two sessions of the HBT: a “no injection” session, and a “CNO injection” session. A within-rat comparison revealed that SI significantly decreased in rats whose NAc was inhibited during acquisition (t(7) = 4.54, *p* = 0.03; Figures 5Q and 5R). No differences in self-grooming or anogenital sniffing were observed (anogenital sniffing, t(7) = 1.01, *p* = 0.35, Figure 5S; self-grooming t(7) = 0.015, *p* = 0.99, Figure 5T). These findings indicate that NAc supports affiliative social behavior during the early stages of the HBT, but it is not necessary for door-opening to occur.

## Discussion

This study explores the neural correlates and role of the oxytocinergic system in prosocial motivation during rat helping behavior, dissociated from social reward. We used an “activity-identity” mapping approach via *c-Fos* and *Oxtr* co-labeling during the SHBT, in which the trapped rat is released into a separate space. This allowed us to determine the neural activity associated with helping trapped ingroup or outgroup members in the absence of social reward as afforded by post-release contact.

### Helping does not depend on post-release contact

We found that of all rats tested, around half became helpers in the SHBT. While this is somewhat below the ratio of helpers reported for the original HBT (∼70% openers^17^), the substantial number of helpers indicates that post-release contact comprises only part of the motivation for door-opening. As helping occurred across strains, sexes, and group memberships, it is not likely that social contact with the released rat was the primary driving factor for door-opening. This finding adds support to previous evidence demonstrating that social contact is not required for helping,^20^^,^^21^^,^^37^^,^^38^ and promotes the idea that rats are motivated by a prosocial intention to improve the trapped rats’ well-being.^39^

### Rats help outgroup members in the separated helping behavior test

We found that in the SHBT, helping occurred, to a non-negligible extent, also toward trapped outgroup members. In previous experiments, helping was only observed toward outgroups in adolescent rats.^19^ This finding suggests that reducing post-release interaction can increase prosocial behavior toward outgroup members in adult rats. The underlying cause of this effect remains unclear. Eliminating contact with the released rat may reduce threat arousal associated with post-release interaction or reduce distress by increasing the distance between the two rats. As helping ingroup members was previously found to reduce corticosterone arousal,^40^ these motivations are difficult to disentangle and should be examined in future studies.

### Dominance and prosocial behavior

The relationship between dominance and prosocial behavior in rodents remains a topic of ongoing debate. While it is commonly assumed that dominant animals are less prosocial, recent experimental studies challenge this notion, revealing that dominant rats and mice often acquire prosocial tendencies faster and select altruistic options more frequently than their subordinate counterparts, particularly as a strategy for group maintenance or status signaling. For example, Gachomba et al.^41^ demonstrated that dominant rats are more likely to display prosocial behavior in response to cues from subordinate cagemates, and Scheggia et al.^42^ found that dominant mice preferentially choose altruistic options in a sharing paradigm. Furthermore, sex hormones such as estrogens and androgens influence both dominance and prosocial behaviors in rodents, modulating social hierarchy formation, aggression, and affiliative interactions.^43^^,^^44^ This hormonal regulation adds another layer of complexity to how social status shapes prosociality in animal groups.

In this study, dominance was not manipulated. However, boldness test results were used as an indirect measure of dominance and hierarchy. While it was previously recorded in the HBT that animals who became openers had lower approach latencies than non-openers to a ledge of an open cage,^39^ in the SHBT, we found no such correlation. These findings suggest that interaction with the trapped rat after helping may mediate the effect of dominance on prosocial behavior. However, this question needs further direct investigation, such as including a Tube Test or a Food Competition Test^45^ before the helping test to determine hierarchy relationships between the free and the trapped rat.

### The prosocial brain network

Quantification of neural activity in the SHBT provided a set of brain regions we termed “the prosocial brain network.” These regions form a dispersed network throughout the brain, encompassing sensory, limbic, frontal, insular, and thalamic areas. However, several regions that showed increased activity in the original HBT (where post-release contact occurs), such as the NAc^18^ were not observed in the SHBT, suggesting that activity in these regions reflects social reward expectation. The NAc, of particular interest, was previously identified as a central hub in the functional connectivity network in the original HBT, and was shown to be active *in vivo* when rats approached a trapped ingroup member.^18^^,^^19^ Here, no increase in NAc activity was observed in the SHBT across experiments. Furthermore, while helping was not prevented by NAc manipulation via chemogenetic excitation or inhibition, affiliative social interaction was reduced during inhibition.

The NAc has been extensively researched in the context of social interaction, and specifically in approach and avoidance behaviors.^46^^,^^47^ However, to our knowledge, few studies have directly used chemogenetic activation or inhibition of the NAc to continuously manipulate social behavior. Most prior research has focused on reward, motivation, or drug-related behaviors,^48^^,^^49^ showing that NAc circuits can strongly influence motivated behaviors. Other studies have highlighted the involvement of various inputs to the NAc in social interactions and parental behaviors, including projections from the VTA,^50^ insula,^51^ and hippocampus.^52^ In voles, chemogenetic inhibition of NAc-sh outputs to the ventral pallidum decreased pair bond formation.^53^ Our findings extend this literature by showing that the chemogenetic inhibition of the NAc itself decreases general social interactions, highlighting a role for NAc circuits in modulating social engagement, even though prosocial helping behavior remains intact.

Together, these findings empirically dissociate social from prosocial motivation at the neural level. While NAc inhibition did not prevent helping, it markedly reduced affiliative social contact, suggesting that the NAc primarily encodes social reward rather than prosocial reinforcement per se.^54^ Notably, a sparse population of NAc-sh neurons remained active and correlated with helping frequency, implying that this subregion may integrate social and prosocial cues under specific conditions.

Theoretically, these results point to a functional distinction between neural circuits that sustain social approach behaviors and those that underlie empathy-driven prosocial motivation. This aligns with cross-species models of empathy, in which subcortical reward circuits—such as the NAc and ventral pallidum—support the hedonic aspects of social engagement, whereas cortical and sensory systems contribute to the affective resonance and perspective-taking components.^8^^,^^55^ By isolating prosocial behavior from social reward, our findings help bridge rodent and primate frameworks for understanding the neural architecture of empathy.

### A role for orbitofrontal cortex in representing others’ outcomes

The OFC was a major player in the prosocial response in the SHBT. This region has also emerged as highly salient during the HBT in previous studies.^18^^,^^19^ The OFC, which is thought to represent valuations of outcomes to the self,^56^^,^^57^^,^^58^ may also assign value to the outcome of others, and thus regulate the prosocial response. Consistent with this idea, the OFC has been highlighted as a hub for decision-making and goal-directed behaviors, as it encodes cognitive maps of the environment,^59^^,^^60^ and valences of relevant cues.^61^^,^^62^^,^^63^

In addition, the OFC receives strong inputs from regions involved in social memory, valuation, categorization, preference, and prosocial behavior, such as the hippocampus, medial prefrontal cortex, lateral septum, and amygdala.^64^^,^^65^^,^^66^^,^^67^^,^^68^^,^^69^^,^^70^^,^^71^^,^^72^^,^^73^ Reflecting these inputs, the OFC has been implicated in coding the value of social outcomes^74^^,^^75^ and in integrating information about social categories.^19^^,^^76^^,^^77^^,^^78^ The significant correlations between OFC activity and helping behavior we found in the SHBT provide further evidence for the central role of the OFC in motivated helping, making this region a promising target for future investigation.

### Differential recruitment of oxytocin receptor*+* cells in sensory versus frontal areas

RNAscope analysis of the *Oxtr* distribution within the ensembles activated during the SHBT revealed a subpopulation of active cells that were OT-sensitive in sensory cortices, but not in frontal regions that were also activated. Although OT involvement cannot be directly concluded from changes in *Oxtr* mRNA transcription levels, there is a body of research conveying the involvement of OT in modulating the sensory processing of social cues, but not non-social cues.^33^^,^^79^^,^^80^^,^^81^ In addition, it has previously been demonstrated that OT plays a critical role in auditory cortex synchronization in response to pup calls, resulting in parental behavior.^25^^,^^27^^,^^82^^,^^83^^,^^84^ One interpretation of the findings presented here is that neural mechanisms underlying empathic arousal involve the oxytocinergic modulation of auditory and visual cortices. However, whether the changes in *Oxtr*+ levels are a direct consequence of OT modulation still needs to be tested.

In conclusion, this study reveals that rats engage in helping behavior even without the possibility of social contact, suggesting prosocial motivation beyond social reward. Neural activity during helping revealed a distributed “prosocial brain network” that recruited oxytocin-sensitive cells in sensory but not frontal cortices. These findings contribute to the understanding of the neurobiological mechanisms underlying empathy and prosocial behavior, providing insight into the possible oxytocinergic modulation processes involved in prosocial motivation, particularly when dissociated from social reward.

### Limitations of the study

The c-Fos data presented in this paper are based on two different methods: immunofluorescence, which measures protein used in experiment 1, and RNAscope, which measures mRNA, used in experiment 2. Discrepancies between these two experiments in the number of active SHBT brain regions may be due to this factor: *c-Fos* mRNA levels are maximally expressed 30 min after the salient event’s onset,^85^ whereas the immunofluorescence data reflect neural activity in the preceding hour. Yet commonalities emerged, and as the emphasis in the RNAscope data was on *Oxtr* mapping, combining these methods has enhanced the results.

Moreover, reliance on c-Fos as a marker for neural activity has several caveats, as we previously described.^18^ Besides having low temporal resolution, c-Fos is an indirect activity indicator and may represent information about plasticity or synchrony along with spiking. Furthermore, the reliance on c-Fos as a neural activity marker allows for only correlational results. In addition, c-Fos is only one out of many immediate-early genes that serve as activity markers in neuroscience. Although these markers generally overlap, some discrepancies exist. Therefore, c-Fos may not be sensitive enough to capture all differences between the tested conditions, which may be visible using other activity markers. Thus, the network presented here should be further investigated using causal manipulations of neural activity.

Another limitation of this study is the small sample size in the RNAscope analysis. Due to limited access to this technology, a subset of the tested animals was quantified for mRNA, providing six animals per condition, males and females combined. Thus, sex differences may have been prevented from emerging.

While our chemogenetic manipulations targeted nucleus accumbens neurons in a general manner, rather than specific cell types or only those activated during helping, we note that such non-specific interventions can still provide valuable insights into the role of this region in motivated behavior. Although the accumbens comprises multiple subpopulations that encode distinct aspects of valence and reward, broad manipulations have been shown to yield meaningful functional outcomes. For example, general inhibition of the NAc is sufficient to suppress amphetamine-induced ultrasonic vocalizations in rats,^86^ and functional clusters of NAc neurons have been shown to span several cell types.^87^ Moreover, recent work demonstrates that valence encoding in the NAc recruits multiple neuronal populations.^88^ Thus, while our non-specific approach may mask the contribution of particular cell populations, it nonetheless informs the necessity and sufficiency of the NAc as a whole in facilitating behavior. At the same time, we recognize the importance of developing more selective tools in rats, which, compared to mice, have a narrower range of transgenic lines available. Activity-dependent targeting methods such as LacZ- and RAM-/CRAM-based systems,^89^^,^^90^ are gaining popularity in rat research and are essential to enable more refined cell-specific targeting in future studies.

## Resource availability

### Lead contact

Further information and requests for resources and reagents should be directed to and will be fulfilled by the lead contact, Prof. Inbal Ben-Ami Bartal (inbalbe@tauex.tau.ac.il).

### Materials availability

This study did not generate any new reagents or animal lines.

### Data and code availability

IHC and RNAscope data have been deposited at OSF and are publicly available as of the date of publication. This paper does not report original code. Any additional information required to reanalyze the data reported in this paper is available from the lead contact upon request.

## Acknowledgments

We thank Dr. Einat Bigelman for assisting with the RNAscope collaboration, Prof. Cornelius Gross, and Prof. Annaliese K Beery for valuable input. We are also grateful to Itsik Sofer, Tamar Spectre, Nesim Gnceer, Aviva Polisar, Annaelle Bismuth, Nir Doron, Or Peleg, Shir Toledano, and Tal Bar-Nahor for their help with data collection. This work was funded by the 10.13039/501100003977Israel Science Foundation (IBB), the 10.13039/501100005155Azrieli Foundation (IBB), and 10.13039/100000874BBRF (IBB).

## Author contributions

Conceptualization, I.B.B. and K.R.; investigation, K.R., E.T., H.F., and A.R.; analysis, I.B.B., K.R., E.T., and B.K.; visualization and writing, I.B.B. and K.R.; methodology, I.B.B., J.K., and A.C.; resources and funding, I.B.B; supervision, I.B.B. and J.K.

## Declaration of interests

The authors declare no competing interests.

## STAR★Methods

### Key resources table

**Table:** 

REAGENT or RESOURCE	SOURCE	IDENTIFIER
**Antibodies**		

Rabbit anti-c-Fos primary antibody	Abcam	Abcam Cat#ab190289; RRID:AB_2737414
Donkey anti-Rabbit IgG (H+L) Highly Cross-Adsorbed Secondary Antibody, Alexa Fluor™ 488	Thermo Fisher Scientific	Thermo Fisher Scientific Cat#A-21206; RRID:AB_2535792

**Bacterial and virus strains**		

pAAV5-hSyn-hM4D(Gi)-mCherry	Addgene	RRID:Addgene_50475
pAAV5-hSyn-hM3D(Gq)-mCherry	Addgene	RRID:Addgene_50474

**Chemicals, peptides, and recombinant proteins**		

Clozapine N-oxide	Tocris Bioscience	Cat#4936

**Deposited data**		

IHC and RNAscope data	This paper	https://osf.io/ve8s7/files/gf7m5

**Experimental models: Organisms/strains**		

Sprague-Dawley Rat	In-house breeding	N/A
Long-Evans Rat	In-house breeding	N/A

**Software and algorithms**		

MATLAB	Mathworks (https://www.mathworks.com)	RRID: SCR_001622
SPSS	IBM	RRID:SCR_019096
GraphPad Prism	GraphPad software http://www.graphpad.com/	RRID: SCR_002798
QuPath	https://qupath.github.io/	RRID:SCR_018257
Cytoscape	https://cytoscape.org/	RRID:SCR_003032
Graph-tool	https://graph-tool.skewed.de/	N/A
Brainways	Kantor et al., 2025 https://github.com/bkntr/brainways	RRID:SCR_024402
EthoVision XT15	Noldus	RRID:SCR_000441
Behavioral Observation Research Interactive Software (BORIS)	Friard and Gamba (2016) https://www.boris.unito.it	RRID:SCR_025700

**Other**		

FISH probe: Fos	ACDBio	Cat No. 403598
FISH probe: OXTr	ACDBio	Cat No. 483678

### Experimental model and study participant details

#### Animals

A total of 24 male Sprague-Dawley (SD) rats, 56 male Long-Evans (LE) rats, and 37 female LE rats were tested as “free” rats across all experiments. All rats started testing at 2-4 months old. Each rat was tested with another same-sex rat as “trapped”, the strain was dependent on the social condition (ingroup = same strain cagemate, outgroup = opposite strain stranger). The two strains were housed in separate rooms and had no contact until the first day of testing (if assigned to the outgroup condition). All rats were socially housed in cages of two same-sex individuals, in a temperature (22-24C) and humidity-controlled (55% relative humidity) animal facility, on a 12:12 light:dark cycle (lights on at 07:00). Food and water were provided *ad libitum*. All testing was done in the rat’s light cycle. All SD rats were ordered from Envigo, Israel, and allowed for at least 2 weeks of acclimation to the vivarium before starting the behavioral procedures. All LE rats were bred in our animal facility and weaned on postnatal day 28. All studies were performed in accordance with protocols approved by the Institutional Animal Ethics Committee at Tel-Aviv University, Israel (approvals #10-21-001, #27133).

### Method details

#### Apparatus

All behavioral procedures were conducted in 52X52X52 plexiglass chambers under light conditions of 80-120 lux. A plexiglass wall (transparent and perforated at the bottom) was used to divide the arena into two equal compartments. All behavioral apparatuses were cleaned with 1% acetic acid and general cleaning soap after every behavioral procedure. All restrainers used across the experiment were identical (25 by 8.75 by 7.5 cm, Mechanical Workshop for Research and Development, School of Chemistry, Tel-Aviv University).

#### Behavioral testing

##### Habituation

Animals underwent five days of habituation, which included daily boldness test sessions followed by 5-minute handling sessions and 30-minute habituation sessions in the arena. During these sessions, the restrainer and door were present in the arena, and the rats could explore the apparatus. On the first day, rats were first tested in an Open Field test (OFT) for 15 minutes instead of the 30-minute habituation to the arena, and only then were they tested for boldness and handled.

##### Open field test (OFT)

Each rat was placed in an empty arena for 20 minutes. Each session was recorded, and the first 15 minutes were analyzed. Movement patterns obtained included total duration spent in the center, latency to first entering the center, frequency of entering the center, mean velocity, and total distance moved.

##### Boldness test

Each cage was tested for 5 minutes for 5 days, and the latency of each rat to rear over the ledge of a halfway-opened cage was documented. In “ingroup” experiments, the boldness test was used to determine who would be the “free” and who would be the “trapped” rat in the HBT by calculating the cumulative latencies of each rat. The rat with the lower cumulative latency was classified as the “bold” rat and, therefore, was determined to be “free”.

##### Helping behavior test (HBT)

Rats were tested in the HBT for 12 daily sessions. Each session was one hour long, during which a free rat was placed in the “separated” arena containing a rat trapped inside a restrainer (Video S1). If the free rat had not opened the restrainer door during the 40 minutes since the beginning of the session, the investigator opened the restrainer door halfway, to a 45° angle; this was typically followed by the exit of the trapped rat and was aimed at preventing learned helplessness. Door-opening was counted as such when performed by the free rat before the halfway opening point. Rats that learned to open the restrainer and consistently opened it on the final three days of testing were labeled as “openers”. Once there was a door-opening, the trapped rat exited to a different compartment and was separated from the free rat by the plexiglass divider. Two social conditions were tested: ingroup and outgroup. In the ingroup condition, the two rats (free and trapped) are from the same strain and have lived together in the same home cage for at least two weeks before the experiment. In the outgroup condition, the two rats are from different strains and were introduced to each other for the first time in the first session of the HBT. The free rat was introduced to the same stranger rat in the restrainer throughout the experiment.

##### HBT with chemogenetic manipulations

The DREADDs’ HBT habituation and testing were similar to the HBT procedure described above. In addition to the regular handling, rats were handled for intraperitoneal (i.p.) injection restraining (with a needle-less syringe). During testing of the first two groups (inhibition n = 8, excitation n = 8), a rat that performed three consecutive days of opening behavior was i.p. injected with Saline 0.9% (3 ml/kg) on the next day to exclude the alternative explanation that stress from the injection will alter the behavior. If opening continued, 3mg/kg Clozapine-N-Oxide (CNO; dissolved in 15ul DMSO 0.5% and filled with saline to injection volume; Cas No: 34233-69-7, Tocris) was i.p. administrated from the next day and every day for at least three days. This dose of CNO was selected as it was shown to be the highest effective and safe from side effects caused by CNO’s reverse-metabolism to clozapine.^91^^,^^92^ After all “openers” were administered with CNO for at least 3 days, all “non-opener” rats were treated with CNO for three additional days. Then, all rats were administered with saline for two final days. All injections were made 40 minutes before the testing session. The third group (inhibition n = 8) received CNO injections on days 1, 3, and 5 of the HBT (independently of opening behavior), with the same dosage as previous groups 40 minutes before the start of the session. On the rest of the days, the rats weren’t given any injections.

##### Social behavior quantification

Ten minutes of social behavior after door-opening were manually coded using BORIS.^93^ Behaviors included General social interaction (sniffing and following), anogenital sniffing, and self-grooming. In the “Acquisition” experiment, the sessions T2 (no injection) and T3 (CNO) were analyzed. In the “Maintenance” experiment, the sessions T1 (no injection), Tn+3 (Saline, 4^th^ day of consecutive opening, Figure 5A), and Tn+4 (1^st^ day of CNO administration) were analyzed.

##### Control sessions for chemogenetic experiments

In order to investigate whether the CNO had a non-specific effect (on non-social/social-related behaviors), several control tests were conducted repeatedly over three days: with saline injection on the first day, CNO injection on the second day, and saline injection again on the third day. The control session included 5 minutes in an empty arena, 5 minutes with four pieces of apple (0.53 cm each) placed in the center of the arena, 5 minutes of interaction with the trapped rat, 5 minutes in an empty arena, 7 minutes with an empty restrainer, and 7 minutes with four pieces of apple placed in a restrainer.

##### Video tracking

All experiments were recorded with a CCD color camera (Sony, China) connected to a video card (Geovision, Taipei, Taiwan) linked to a PC. Movement data were analyzed using EthovisionXT15 video tracking software (Noldus Information Technology, Inc, Leesburg, VA).

#### Stereotaxic surgeries

Male LE rats (age 2-4 months) underwent stereotaxic injection of a viral vector containing an inhibitory (AAV8-hSyn-hM4D(Gi)-mCherry, n = 16; Addgene #50475) or an excitatory (AAV-hSynhM3D(Gq)-mCherry, n = 8; Addgene #50474) Designer Receptors Exclusively Activated by Designer Drugs (DREADDs) to the NAc (coordinates: A/P 1.5, M/L ±1.5, D/V -8.0). Rats were anesthetized with isoflurane (induction: 3-5%, maintenance: 1-3%) and mounted onto a stereotaxic frame. The skull was exposed following subcutaneous (s.c.) injection of Lidocaine (5mg/kg, 2%), and two small holes were made above the determined stereotaxic coordinates. A Hamilton Syringe with a needle of 33g containing the virus was used to inject 0.89 ul in each hemisphere. Following surgery, the rats were given s.c. injections of pain relievers (1mg/kg of Meloxicam 0.5%, 0.05mg/kg of Buprenorphine 0.3mg/ml) and Saline (10ml/kg) to ensure hydration. The rats were allowed for two weeks to recover and then started the habituation and the HBT protocol, allowing three weeks in total for viral expression.

##### Virus injection validation

After testing in the HBT and control sessions, rats underwent one final day of HBT with a latched restrainer as described in the former HBT procedure, after which the rats were sacrificed, and brains were obtained after perfusions with 1XPBS and 4% paraformaldehyde. Brains were cryosectioned in 40μm and stained for c-Fos (rabbit anti-c-Fos, ab190289, Abcam; Alexa Fluor 488-conjugated donkey anti-rabbit, A-21206, Thermo Fischer) and DAPI as described in the “Immunofluorescence” section above. Co-labeling of c-Fos and mCherry was manually quantified in a representative area of 250μm^2^ from the NAc from each rat.

#### Immunofluorescence stainings

At the end of the HBT, the SD rats underwent one additional final session with a latched restrainer and were sacrificed within 60 minutes from the beginning of the session, at the peak expression of the immediate-early-gene c-Fos. Rats were transcardially perfused with 1XPBS and freshly made 4% paraformaldehyde (PFA). Brains were then kept in 4% PFA overnight, sunk in 30% sucrose for an additional 48 hours or until sunk, and stored at -80°C. They were later coronally sliced at 40μm (Leica CM3050 S; Leica 819 Low Profile Microtome Blades) and stored in cryoprotectant (Sucrose, Polyvinyl-pyrrolidone (PVP-40), 0.1M PB, Ethylene glycol) until stained for c-Fos. Free-floating sections were washed with 0.1M tris-buffered saline (TBS, 3×5′), incubated for 1 hour in 3% normal donkey serum (NDS) in 0.3% Triton X-100 in TBS (tTBS), and then transferred to rabbit anti-c-Fos (ab190289, Abcam, 1:1000; 1% NDS; 0.3% tTBS) in 4°C overnight. Sections were then washed in 0.1 M TBS (3×5′) and incubated for 2 hours in Alexa Fluor 488-conjugated donkey anti-rabbit (A-21206, 1:1000; 1% NDS; 0.3% tTBS). Sections were briefly washed in 0.1M TBS again (3×5′) and further stained in DAPI (1:40,000) for 10 min, then washed for an additional 15 min (3×5′). Lastly, all slides were mounted and coverslipped with 2.5% PVA/DABCO, dried overnight, and stored at 4°C until imaged. Immunostained tissue was imaged at 10× using a wide-field fluorescence microscope (Olympus ix83) and was processed (registered to rat brain atlas and quantified) in our open-source software Brainways.^22^

#### Multiplex RNAscope

Brains from LE rats tested in the “separated” HBT in each social condition described above were used for fluorescent *in situ* hybridization (ISH) essays: ingroup n = 8 (5 males), outgroup n = 6 (3 males), and naive baseline n = 6 (3 males). One rat in the baseline condition was found to be an outlier and was excluded, resulting in n = 5 in this group. All rats in the ingroup condition were openers except for 2 males, and all rats in the outgroup condition were non-openers. Animals were selected for this analysis based on consistent behavioral performance in the SHBT as well as high-quality perfusion and tissue integrity. As above, in the final session, restrainers were latched so that neural activity reflects an hour in the presence of a trapped rat. Rats were transcardially perfused with 1XPBS after the last HBT session. The brains were then snap-frozen and stored at -80°C until cryosectioning at 18μm. Coronal slices were collected on Superfrost Plus slides (Thermo Scientific) from each brain (4.20mm, 1.56mm, 0.00mm, -2.76mm, -3.36mm, -4.44mm, and -5.64mm from bregma) and underwent processing for single molecule fluorescent *in situ* hybridization (smFISH) assay. Slides were pre-treated with RNAscope Protease III reagent. smFISH was performed on slides using the RNAscope LS Multiplex Reagent Kit (Advanced Cell Diagnostics) and LS 4-Plex Ancillary Kit and Multiplex Reagent Kit on a robotic staining system (Leica BONDIII). RNAscope probes were *Fos* (ACD, 403598), and *Oxtr* (ACD, 483678). Images were acquired on a Vectra Polaris Automated Quantitative Pathology Imaging System (Akoya Biosciences) at 20x magnification. Cellular and subcellular detection was conducted using QuPath software and implemented into Brainways for image registration and brain-wide quantification. Due to the limited parcellation of the hypothalamic subregions in the Waxholm rat brain atlas, the medial preoptic nucleus (MPON; bregma -0.24), paraventricular nucleus (PVN; Bregma -1.8), and Ventromedial nucleus (VMH; bregma -2.4) were manually identified and quantified in QuPath (see Figure S3A for a representative manual parcellation).

### Quantification and statistical analyses

Statistical analyses were performed using IBM SPSS Statistics version 28.0.1.0 (142) and GraphPad Prism 9. All means are reported as mean±SEM. Statistical significance in figures is indicated using asterisks, with p < 0.05 (∗), p < 0.01 (∗∗), p < 0.001 (∗∗∗), and p < 0.0001 (∗∗∗∗), unless otherwise stated in the figure legend. Exact p-values and the statistical tests used are reported in the results section and figure legends. Depending on the experimental design, data were analyzed using parametric or nonparametric tests, including two-tailed unpaired or paired t-tests (with Welch’s correction when variances were unequal), one-way or two-way ANOVA (including repeated-measures designs where appropriate), mixed-model ANOVA (MMA), Friedman tests for repeated nonparametric measures, Cochran’s Q tests for repeated binary outcomes, and Fisher’s exact tests for categorical data. Post hoc comparisons were performed using Tukey’s or Bonferroni’s multiple comparisons tests as specified. Correlation analyses were conducted using Pearson’s or Spearman’s correlation coefficients, with test selection based on sample size and data distribution. For brain-wide analyses involving multiple regions, false discovery rate (FDR) correction was applied using the two-stage linear step-up procedure of Benjamini, Krieger, and Yekutieli, with adjusted significance thresholds indicated in the results and figure legends.

Network graphs were generated by first obtaining a correlation matrix of c-Fos activity between all brain regions (using pairwise Pearson or Spearman’s correlation coefficients). All significant correlations (p < 0.05) were presented in a graphic form using Cytoscape.^94^ Correlation with significance values higher than the cutoff were set to one and the corresponding brain regions greater than 1 were considered connected to the network. Central hubs were identified as regions scoring in the top 20% for both degree (the number of connections) and betweenness (representing how many regions connect to others through this region. Nodes parameters (degree, centrality) were analyzed with Cytoscape.
