# Supplementary material for: Dissociating neural circuits of social and prosocial reward in rat helping behavior
Source: iScience. 2026 Jan 14;29(2):114694. doi: 10.1016/j.isci.2026.114694 (PMC12874461; doi:10.1016/j.isci.2026.114694)
Supplement: Document S1. Figures S1–S5 and Tables S1–S4 [file mmc1.pdf]

iScience, Volume *29*

Supplemental information

Dissociating neural circuits of social and prosocial reward in rat helping behavior

Keren Ruzal, Estherina Trachtenberg, Ben Kantor, Hila Flumin, Adin Roemer, Andres Crespo, Johannes Kohl, and Inbal Ben-Ami Bartal

Document S1: supplementary information

Supplementary Figure legends

Fig. S1. Open Field Test and Boldness test results for all behavioral experiments. a-f) Male SD rats. **g-l)** Male LE rats. **p-r)** Female LE rats.

Fig. S2. Brain-wide c-Fos quantification via immunofluorescence staining per region and per social condition.

Fig. S3. Supplementary analyses for the brain-wide c-Fos mapping in the SHBT. a) Representative images of manual parcellation of the hypothalamic subregions: medial preoptic nucleus (MPON), paraventricular nucleus (PVN), and Ventromedial nucleus (VMH). **b)** Brain-wide c-Fos levels show no brain-wide difference between openers and non-openers (ANOVA main effect, $F(2, 21) = 6.494$, $p = 0.006$; Tukey's multiple comparisons test, openers vs. non-openers, $p = 0.19$). **b)** Brain-wide comparison of c-Fos levels between openers and non-openers tested in the HBT and the SHBT shows different activity patterns, in a paradigm- and behavior-dependent manner. **c)** Louvain clustered heatmap of pairwise correlation values for the SHBT. The bars on the left visualize the identified clusters and regions' categories.

Fig. S4. Viral spread in NAc chemogenetic experiments. a) Experiment 1: Experiment 1: NAc inhibition after opening has been learned. **b)** Experiment 2: NAc excitation after opening has been learned. **c)** Experiment 3: Inhibition at the beginning of HBT.

Fig. S5. Timeline and control tests of NAc CNO-induced chemogenetic manipulation. a-b) Co-labeling of the viral-infected cells with c-Fos as a validation of the chemogenetic manipulation via CNO administration. **c-d)** All rats received a saline i.p injection once reaching the criterion of 3 consecutive openings, followed by at least 3 days of CNO

injections. In the last days of HBT all rats received additional injections of saline prior to each session. **e)** HBT results for the excitation experiment: NAc excitation did not impede nor facilitate door opening or activity levels in the HBT. **f-g)** Results of control tests conducted at the end of the HBT in the inhibition (f) and excitation (g) experiments. Appearance of the results is according to the testing order. CNO administration did not significantly affect locomotor activity and anxiety, non-social reward-seeking or consumption, interest in the restrainer, or general social interaction.

Fig. S1

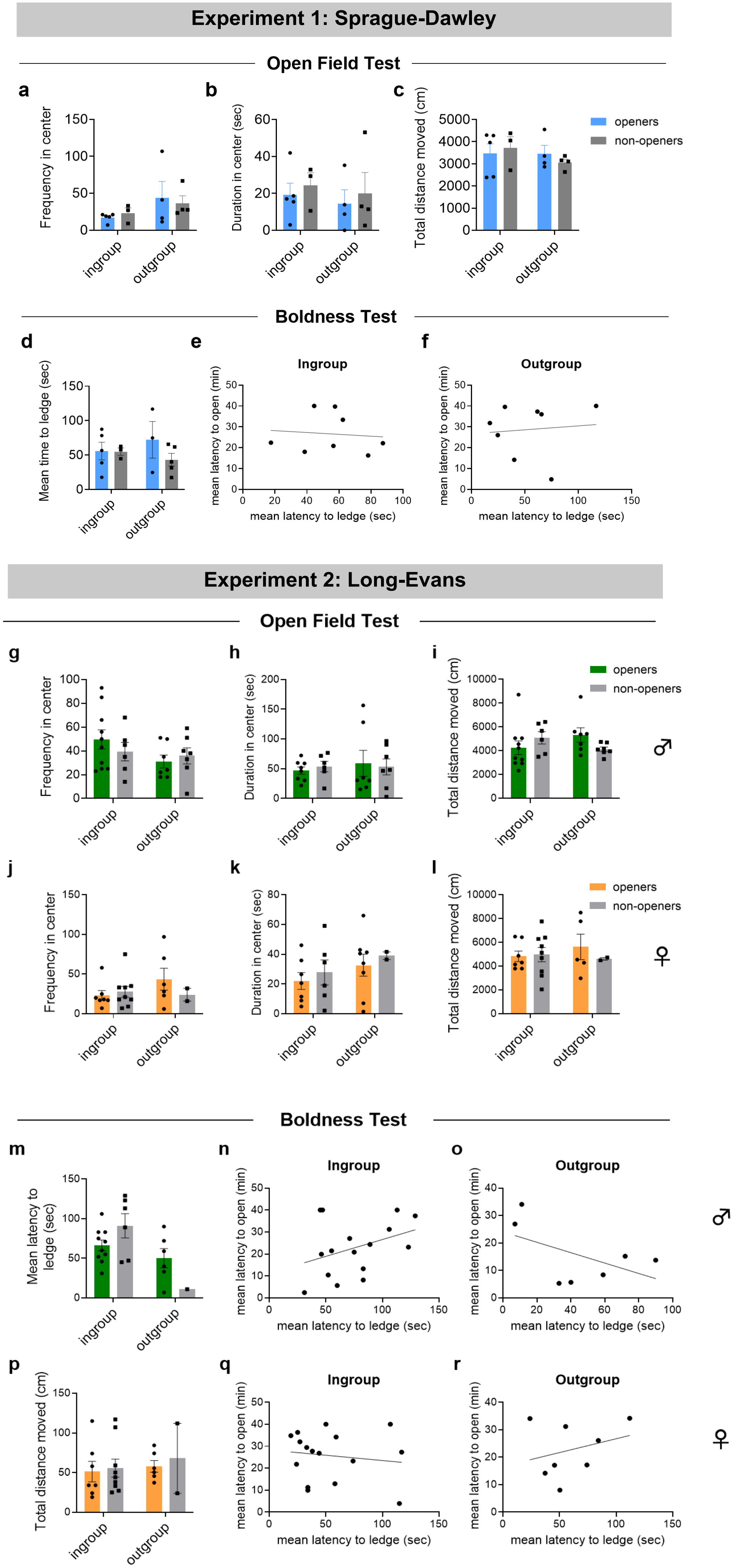

Experiment 1: Sprague-Dawley
Open Field Test
a
Frequency in center
b
Duration in center (sec)
c
Total distance moved (cm)
openers
non-openers
ingroup
outgroup
Boldness Test
d
Mean time to ledge (sec)
e
Ingroup
mean latency to open (min)
mean latency to ledge (sec)
f
Outgroup
Experiment 2: Long-Evans
Open Field Test
g
h
i
j
k
l
♂
♀
Boldness Test
m
Mean latency to ledge (sec)
n
o
p
q
r

Fig. S2

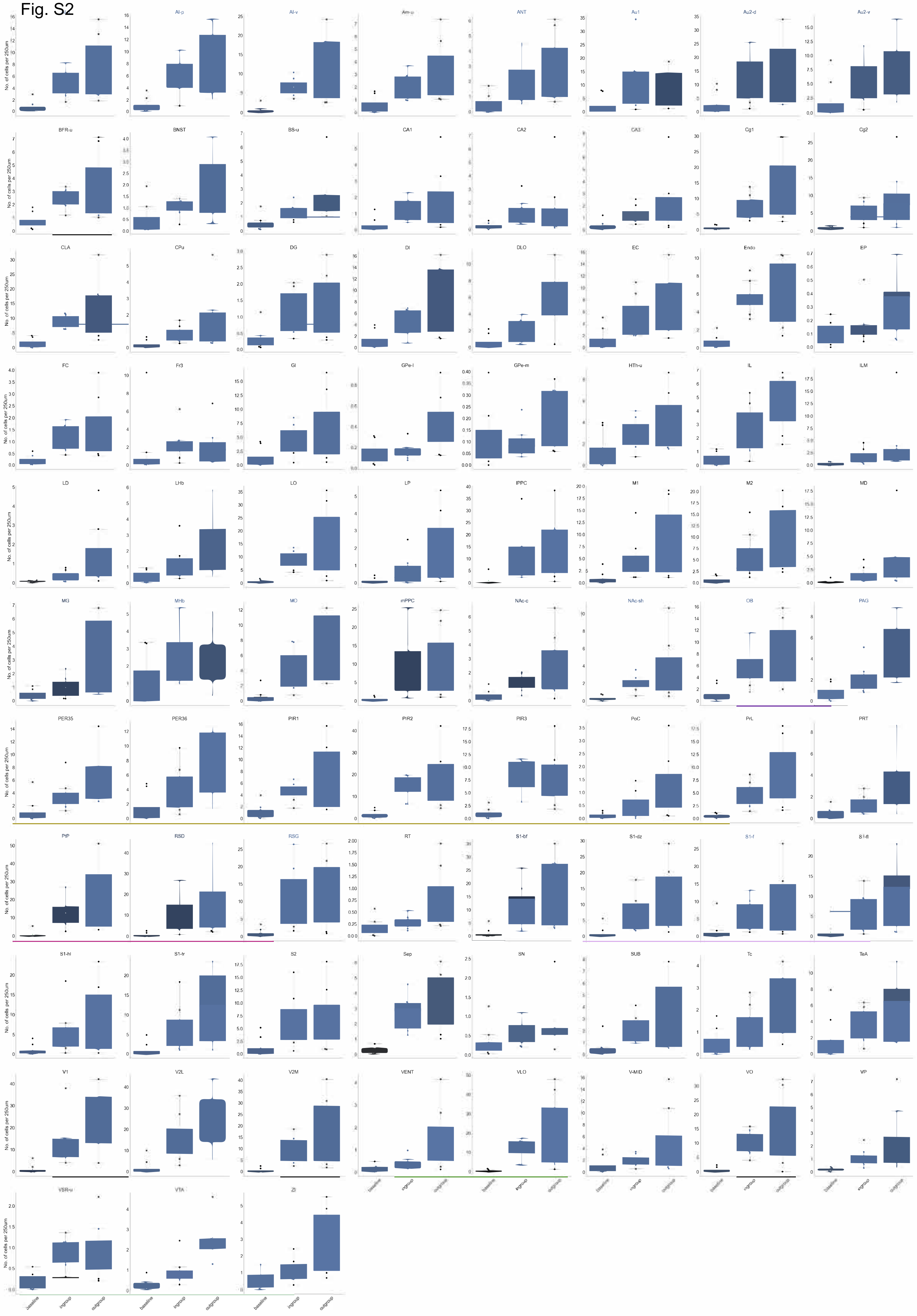
No. of cells per 250um
AI-p
AI-v
Am-u
ANT
Au1
Au2-d
Au2-v
BFR-u
BNST
BS-u
CA1
CA2
CA3
Cg1
Cg2
CLA
CPu
DG
DI
DLO
EC
Endo
EP
FC
Fr3
GI
GPe-l
GPe-m
HTh-u
IL
ILM
LD
LHb
LO
LP
lPPC
M1
M2
MD
MG
MHb
MO
mPPC
NAc-c
NAc-sh
OB
PAG
PER35
PER36
PIR1
PIR2
PIR3
PoC
PrL
PRT
PtP
RSD
RSG
RT
S1-bf
S1-dz
S1-f
S1-fl
S1-hl
S1-tr
S2
Sep
SN
SUB
Tc
TeA
V1
V2L
V2M
VENT
VLO
V-MID
VO
VP
VSR-u
VTA
ZI
baseline
ingroup
outgroup

Fig. S3

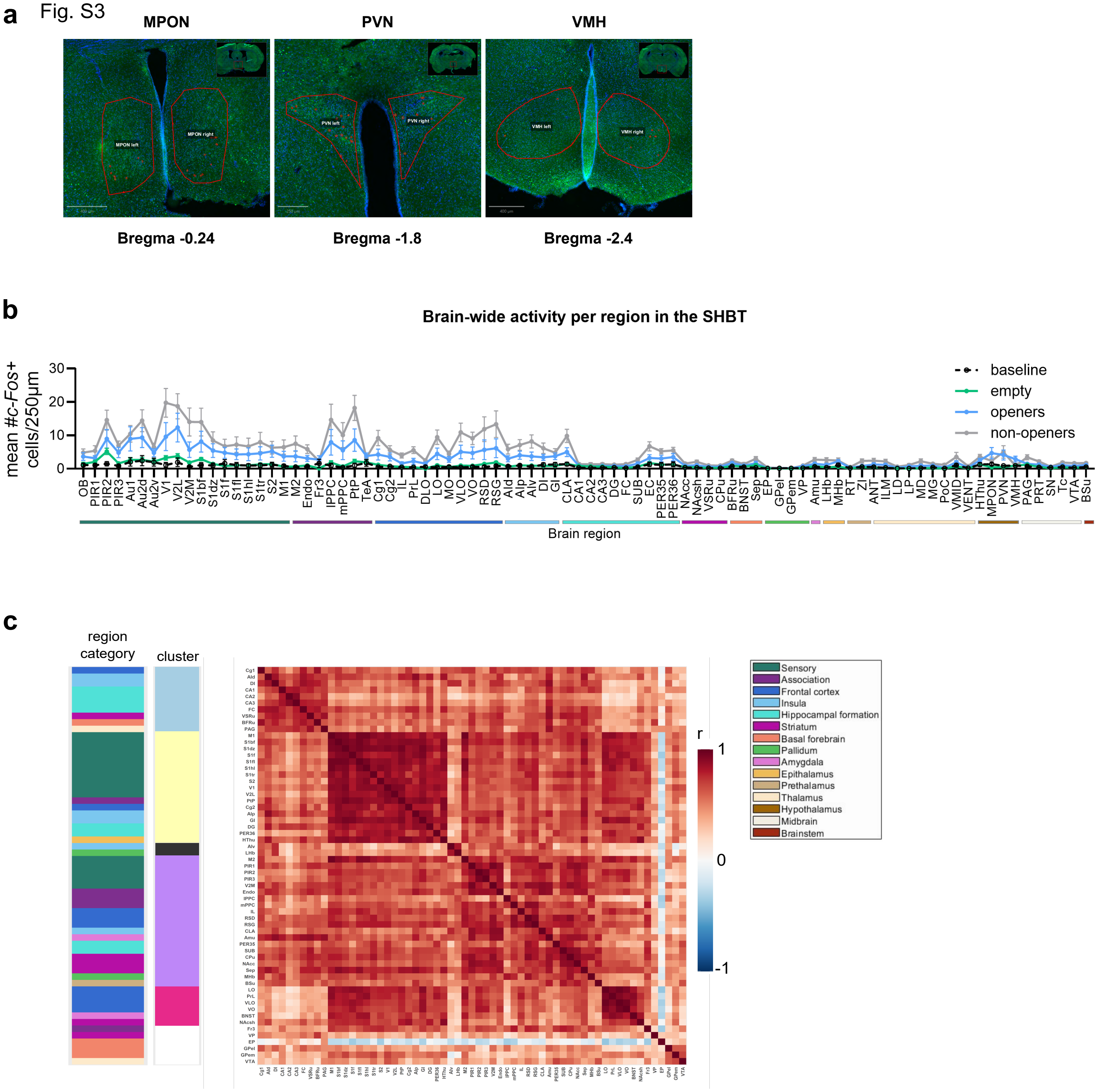
a
MPON
PVN
VMH
MPON left
MPON right
PVN left
PVN right
VMH left
VMH right
Bregma -0.24
Bregma -1.8
Bregma -2.4
b
Brain-wide activity per region in the SHBT
mean #c-Fos+ cells/250µm
baseline
empty
openers
non-openers
Brain region
c
region category
cluster
r
1
0
-1
Sensory
Association
Frontal cortex
Insula
Hippocampal formation
Striatum
Basal forebrain
Pallidum
Amygdala
Epithalamus
Prethalamus
Thalamus
Hypothalamus
Midbrain
Brainstem

Fig. S4

DREADDs manipulation in NAc - viral spread summary

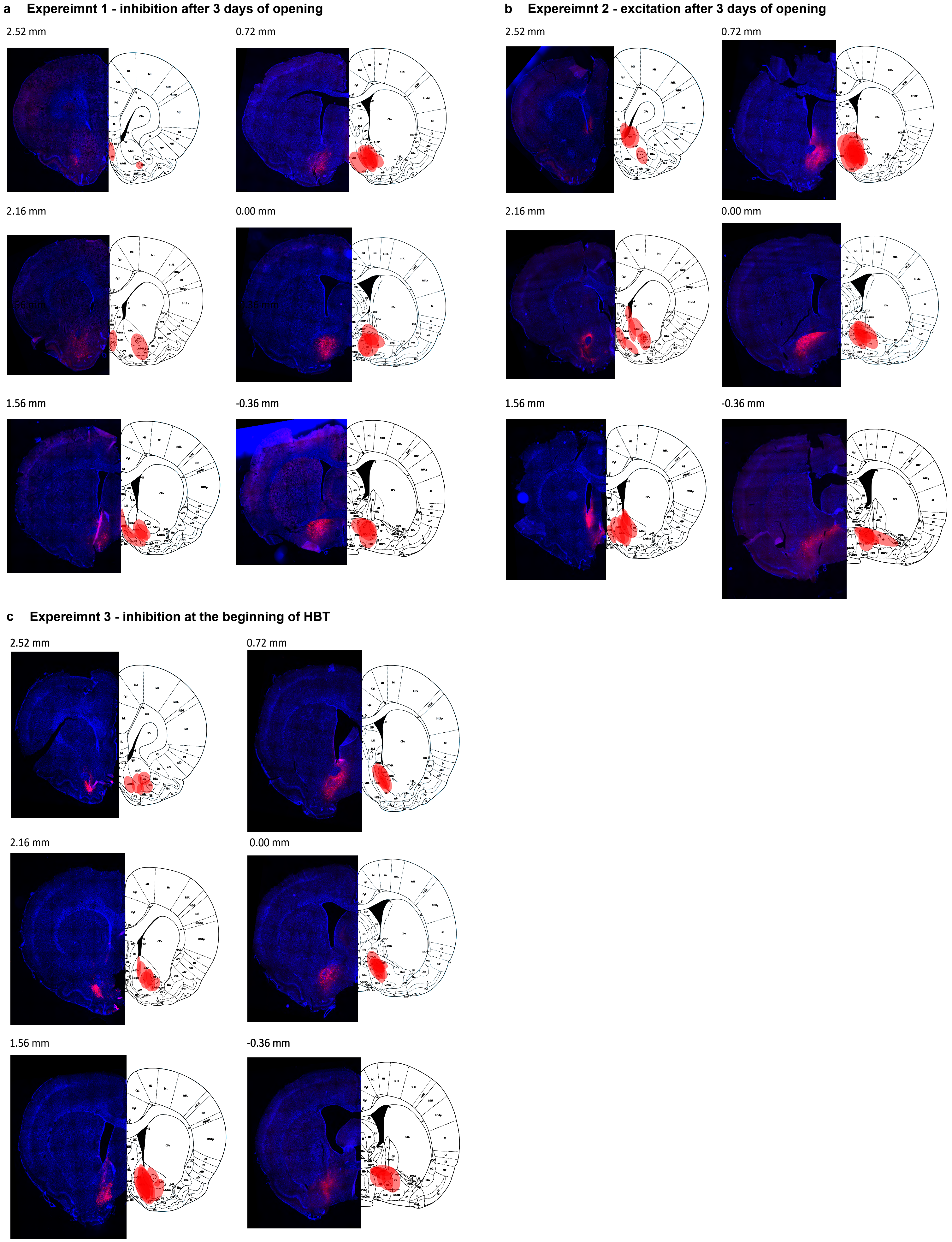

Fig. S5

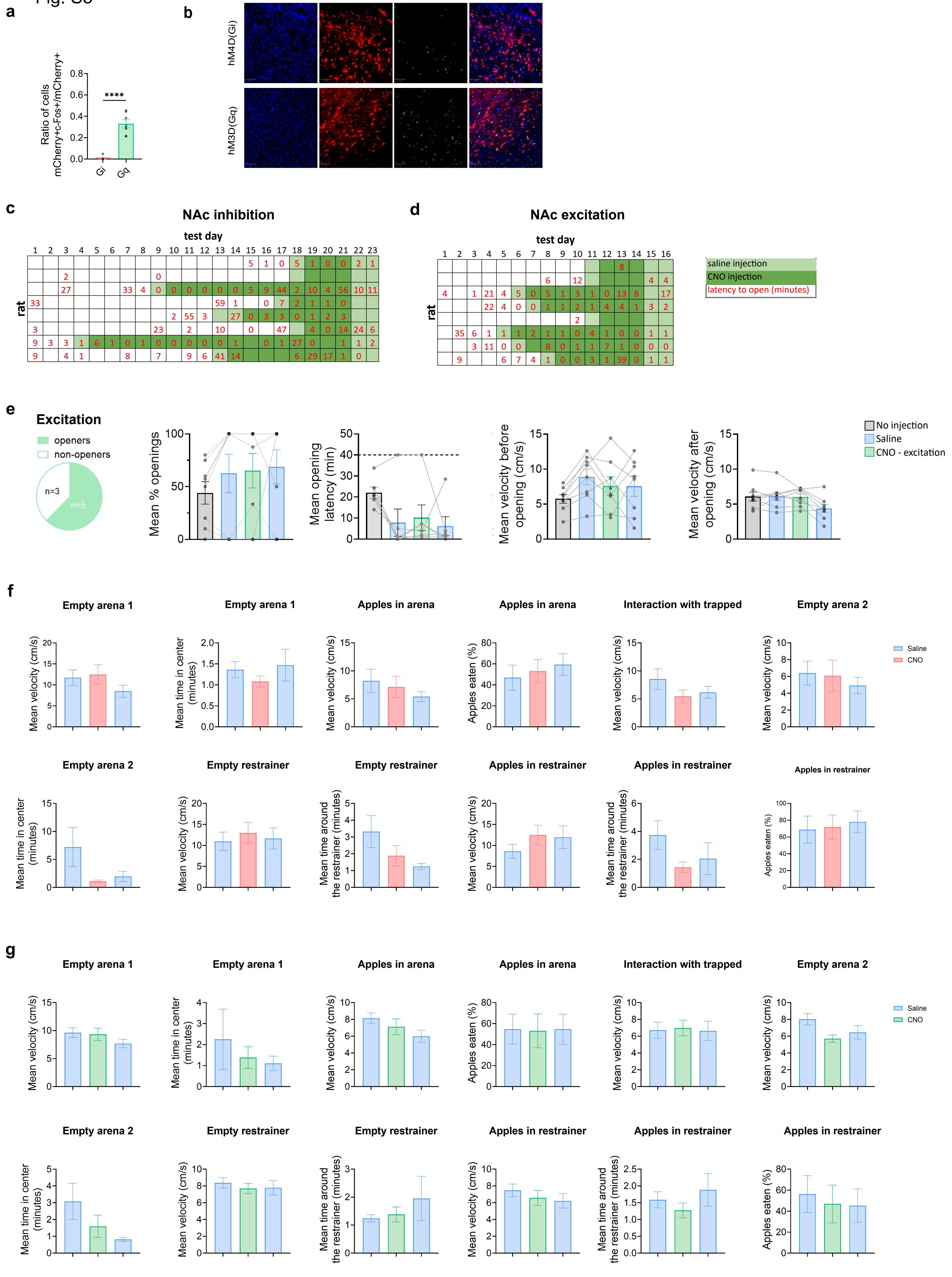
a
Ratio of cells mCherry+c-Fos+/mCherry+
****
Gi
Gq
b
DAPI
mCherry
c-Fos
Merged
hM4D(Gi)
hM3D(Gq)
c
NAc inhibition
test day
rat
d
NAc excitation
test day
rat
saline injection
CNO injection
latency to open (minutes)
e
Excitation
openers
non-openers
n=3
n=5
Mean % openings
Mean opening latency (min)
Mean velocity before opening (cm/s)
Mean velocity after opening (cm/s)
No injection
Saline
CNO - excitation
f
Empty arena 1
Empty arena 1
Apples in arena
Apples in arena
Interaction with trapped
Empty arena 2
Saline
CNO
Empty arena 2
Empty restrainer
Empty restrainer
Apples in restrainer
Apples in restrainer
Apples in restrainer
Mean velocity (cm/s)
Mean time in center (minutes)
Apples eaten (%)
Mean time around the restrainer (minutes)
g
Empty arena 1
Empty arena 1
Apples in arena
Apples in arena
Interaction with trapped
Empty arena 2
Saline
CNO
Empty arena 2
Empty restrainer
Empty restrainer
Apples in restrainer
Apples in restrainer
Apples in restrainer

Tables

Table S1. List of brain regions analyzed and used throughout the paper.

acronym	name
AId	Agranular insular cortex dorsal area
AIp	Agranular insular cortex, posterior area
AIv	Agranular insular cortex, ventral area
Amu	Amygdaloid area, unspecified
ANT	Anterior nuclei of the dorsal thalamus
Au1	Primary auditory area
Au2d	Secondary auditory area, dorsal part
Au2v	Secondary auditory area, ventral part
BFRu	Basal forebrain region, unspecified
BNST	Bed nucleus of the stria terminalis
BSu	Brainstem, unspecified
CA1	Cornu ammonis 1
CA2	Cornu ammonis 2
CA3	Cornu ammonis 3
Cg1	Cingulate area 1
Cg2	Cingulate area 2
CL	Central lateral thalamic nucleus
CLA	Claustrum
CPu	Caudate putamen
DG	Dentate gyrus
DI	Dysgranular insular cortex
DLG	Dorsal lateral geniculate nucleus
DLO	Dorsolateral orbital area
Endo	Endopiriform nucleus
EP	Entopeduncular nucleus
Eth	Ethmoid-Limitans nucleus
FC	Fasciola cinereum
Fr3	Frontal association area 3
GI	Granular insular cortex
GPel	Globus pallidus external, lateral part
GPem	Globus pallidus external, medial part
HThu	Hypothalamic region, unspecified
IAM	Interanteromedial thalamic nucleus

IGL	Intergeniculate leaflet
IL	Infralimbic area
IMD	Intermediodorsal thalamic nucleus
LDdm	Laterodorsal thalamic nucleus, dorsomedial part
LDvl	Laterodorsal thalamic nucleus, ventrolateral part
LEC	Lateral entorhinal cortex
LHb	Lateral habenular nucleus
LO	Lateral orbital area
LP	Lateral posterior thalamic nucleus
lPPC	Parietal association cortex, lateral area
M1	Primary motor area
M2	Secondary motor area
MD	Mediodorsal thalamic
MEC	Medial entorhinal cortex
MG	Medial geniculate body
MHb	Medial habenular nucleus
MO	Medial orbital area
mPPC	Parietal association cortex, medial area
NAcc	Nucleus accumbens, core
NAcsh	Nucleus accumbens, shell
NLOT	Nucleus of the lateral olfactory tract
OBu	Olfactory bulb, unspecified
PAG	Periaqueductal gray
PER35	Perirhinal area 35
PER36	Perirhinal area 36
PIR1	Piriform cortex, layer 1
PIR2	Piriform cortex, layer 2
PIR3	Piriform cortex, layer 3
Po	Posterior thalamic nucleus
Pot	Posterior thalamic nuclear group, triangular part
PP	Peripeduncular nucleus
PrG	Pregeniculate nucleus
PrL	Prelimbic area
PrS	Presubiculum
PRT	Pretectal region
RT	Reticular (pre)thalamic nucleus
PT	Parataenial thalamic nucleus
PtP	Parietal association cortex, posterior area

PV	Paraventricular thalamic nuclei (anterior and posterior)
RSD	Retrosplenial dysgranular area
RSG	Retrosplenial granular area
RTa	Reticular (pre)thalamic nucleus, auditory segment
RTu	Reticular (pre)thalamic nucleus, unspecified
S1bf	Primary somatosensory area, barrel field
S1dz	Primary somatosensory area, dysgranular zone
S1f	Primary somatosensory area, face representation
S1fl	Primary somatosensory area, forelimb representation
S1hl	Primary somatosensory area, hindlimb representation
S1tr	Primary somatosensory area, trunk representation
S2	Secondary somatosensory area
Sep	Septal region
SMn	Nucleus of the stria medullaris
SMT	Submedius thalamic nucleus
SN	Substantia nigra
SUB	Subiculum
SubG	Subgeniculate nucleus
SuD	Deeper layers of the superior colliculus
SuG	Superficial gray layer of the superior colliculus
TeA	Temporal association cortex
V1	Primary visual area
V2L	Secondary visual area, lateral part
V2M	Secondary visual area, medial part
VA	Ventral anterior thalamic nucleus
VENT	Ventral nuclei of the dorsal thalamus
VL	Ventrolateral thalamic nucleus
VLO	Ventrolateral orbital area
VM	Ventromedial thalamic nucleus
VMID	Ventral midline group of the dorsal thalamus
VO	Ventral orbital area
VP	Ventral pallidum
VPL	Ventral posterolateral thalamic nucleus
VPM	Ventral posteromedial thalamic nucleus
VPpc	Ventral posterior nucleus of the thalamus, parvicellular part
VSRu	Ventral striatal region, unspecified
VTA	Ventral tegmental area
ZI	Zona incerta

Table S2. P values of baseline vs. SHBT IHC c-Fos cell counts t-tests with Two-stage linear step-up procedure of Benjamini, Krieger and Yekutieli method for FDR correction (Q=1%, threshold: $p < 0.006$; $q < 0.01$).

Region	Discovery?	P value	q value	Cohen's d	t value
OB	Yes	0.0030	0.0070	3.8467	-3.3037
PIR1	Yes	0.0030	0.0070	3.5206	-3.3564
PIR2	Yes	0.0000	0.0000	7.9263	-4.4276
PIR3	Yes	0.0000	0.0000	3.5267	-4.7090
Au1	No	0.0140	0.0150	7.3581	-2.6682
Au2d	Yes	0.0040	0.0080	8.1059	-3.2208
Au2v	No	0.0140	0.0150	3.8728	-2.6684
V1	Yes	0.0010	0.0050	9.8995	-4.0532
V2L	Yes	0.0000	0.0000	9.7599	-4.1130
V2M	Yes	0.0030	0.0070	9.6942	-3.3207
S1bf	Yes	0.0020	0.0060	8.9160	-3.5411
S1dz	No	0.0070	0.0110	6.7341	-2.9630
S1f	No	0.0250	0.0240	6.1573	-2.3971
S1fl	No	0.0100	0.0130	5.7450	-2.8115
S1hl	No	0.0160	0.0170	6.0212	-2.6058
S1tr	No	0.0140	0.0150	6.5693	-2.6647
S2	Yes	0.0060	0.0100	4.5096	-3.0107
M1	No	0.0090	0.0130	5.2388	-2.8689
M2	Yes	0.0020	0.0060	5.2693	-3.4681
Endo	Yes	0.0000	0.0000	2.3668	-5.1307
Fr3	No	0.5320	0.3730	2.5588	-0.6350
lPPC	Yes	0.0050	0.0090	9.2792	-3.1482
mPPC	Yes	0.0040	0.0080	6.7480	-3.2067
PtP	Yes	0.0010	0.0050	10.5865	-3.7595
TeA	No	0.0580	0.0470	2.7763	-1.9995
Cg1	Yes	0.0020	0.0060	7.1739	-3.4428
Cg2	No	0.0080	0.0120	5.2160	-2.9003
IL	Yes	0.0000	0.0000	1.7382	-4.2407
PrL	Yes	0.0030	0.0070	4.1800	-3.3906
DLO	No	0.0120	0.0140	2.3123	-2.7264
LO	Yes	0.0030	0.0070	7.9622	-3.3588
MO	Yes	0.0010	0.0050	2.7045	-3.9086
VLO	Yes	0.0040	0.0080	10.9475	-3.2139
VO	Yes	0.0010	0.0050	7.2492	-3.7016
RSD	No	0.0080	0.0120	9.9358	-2.9058
RSG	Yes	0.0030	0.0070	7.5560	-3.3427
AId	Yes	0.0020	0.0060	3.4148	-3.5505
AIp	Yes	0.0010	0.0050	3.7087	-3.6927
AIv	Yes	0.0020	0.0060	5.2945	-3.5135
DI	No	0.0100	0.0130	4.0042	-2.8411
GI	No	0.0160	0.0170	3.8624	-2.6077
CLA	Yes	0.0020	0.0060	6.0918	-3.5000

CA1	No	0.0260	0.0250	1.1930	-2.3876
CA2	No	0.0570	0.0470	1.3966	-2.0094
CA3	No	0.0500	0.0430	1.5099	-2.0774
DG	No	0.0090	0.0130	0.6918	-2.8589
FC	Yes	0.0020	0.0060	0.7940	-3.4444
SUB	Yes	0.0050	0.0090	1.6974	-3.1133
EC	Yes	0.0050	0.0090	3.4169	-3.0903
PER35	Yes	0.0060	0.0100	2.9321	-3.0749
PER36	No	0.0100	0.0130	3.2066	-2.8137
NAcc	No	0.0140	0.0150	1.4036	-2.6553
NAcsh	No	0.0110	0.0140	2.1562	-2.7653
VSRu	Yes	0.0010	0.0050	0.4322	-3.8311
CPu	No	0.0350	0.0320	1.1145	-2.2455
BFRu	Yes	0.0020	0.0060	1.5249	-3.4662
BNST	No	0.0220	0.0220	0.9646	-2.4611
Sep	Yes	0.0000	0.0000	1.3018	-5.1091
EP	No	0.0520	0.0440	0.1596	-2.0582
GPel	No	0.0810	0.0630	0.1990	-1.8264
GPem	No	0.4280	0.3070	0.1169	-0.8082
VP	No	0.0340	0.0310	1.5144	-2.2623
Amu	Yes	0.0040	0.0080	1.5401	-3.2094
LHb	No	0.0100	0.0130	1.2896	-2.7980
MHb	No	0.0660	0.0530	1.5056	-1.9308
RT	No	0.0730	0.0570	0.4291	-1.8822
ZI	No	0.0240	0.0230	1.3686	-2.4231
ANT	No	0.0110	0.0140	1.6010	-2.7761
ILM	No	0.1050	0.0770	3.6462	-1.6891
LD	No	0.0700	0.0560	1.0325	-1.9013
LP	No	0.0480	0.0420	1.3265	-2.0902
MD	No	0.0940	0.0720	3.5232	-1.7483
MG	No	0.0440	0.0390	1.5651	-2.1399
PoC	No	0.0290	0.0270	0.8027	-2.3370
VMID	No	0.1000	0.0740	3.4819	-1.7186
VENT	No	0.0520	0.0440	0.9115	-2.0527
HThu	No	0.0120	0.0140	1.9749	-2.7498
MPON	No	0.4770	0.3380	11.6801	-0.7370
PVN	No	0.3520	0.2560	49.9794	-0.9644
VMH	No	0.7120	0.4930	5.4367	-0.3764
PAG	Yes	0.0060	0.0100	1.8565	-3.0610
PRT	No	0.0200	0.0200	1.6694	-2.5129
SN	No	0.0960	0.0720	0.4879	-1.7412
Tc	No	0.0180	0.0180	1.0593	-2.5588
VTA	Yes	0.0020	0.0060	0.8965	-3.4372
BSu	No	0.0120	0.0140	1.2006	-2.7256

Table S3. Statistics of baseline vs. SHBT RNAscope *c-Fos* cell counts t-tests with Two-stage linear step-up procedure of Benjamini, Krieger and Yekutieli method for FDR correction (Q=1%, threshold: $p < 0.00025$).

Region	Discovery?	P value	q value	Cohen's d	t value
OB	No	0.174	0.59178	5.83065	-1.426
PIR1	No	0.312	0.63832	3.66872	-1.042
PIR2	No	0.216	0.59344	5.5177	-1.285
PIR3	No	0.498	0.76488	2.65795	-0.692
Au1	Yes	0.0001	0.00399	3.14037	-4.216
Au2d	Yes	0.0001	0.00399	2.59183	-4.273
Au2v	No	0.003	0.04787	3.21267	-3.429
V1	No	0.082	0.40892	5.6157	-1.873
V2L	No	0.147	0.58646	3.12478	-1.542
V2M	No	0.455	0.76488	3.70036	-0.765
S1bf	No	0.981	0.96635	3.8901	0.025
S1dz	No	0.89	0.93438	4.62653	-0.141
S1f	No	0.763	0.85746	5.66426	-0.306
S1fl	No	0.54	0.76488	5.54122	-0.627
S1hl	No	0.284	0.63832	5.07468	-1.122
S1tr	No	0.674	0.80494	2.52283	0.428
S2	No	0.757	0.85746	3.25018	0.315
M1	No	0.308	0.63832	4.53367	-1.053
M2	No	0.378	0.70141	4.77358	-0.906
lPPC	No	0.949	0.95849	3.81999	-0.065
mPPC	No	0.678	0.80494	4.26911	-0.423
PtP	No	0.002	0.0399	3.2016	-3.798
TeA	No	0.002	0.0399	2.94287	-3.935
Endo	No	0.334	0.65	2.31636	-0.994
Cg1	No	0.555	0.76488	7.32873	-0.603
Cg2	No	0.717	0.82912	4.81617	-0.369
MFC	No	0.077	0.40892	5.3425	-1.906
LO	No	0.014	0.13963	9.18489	-2.962
VLO	No	0.01	0.11399	9.88055	-3.116
VO	No	0.006	0.07979	8.30591	-3.445
RSD	No	0.479	0.76488	4.40411	0.724
RSG	No	0.105	0.46544	3.41262	-1.711
AId	No	0.04	0.26597	5.50835	-2.25
AIp	No	0.838	0.9068	2.72708	0.207
AIv	No	0.214	0.59344	4.64347	-1.296
DI	No	0.782	0.86661	3.0554	0.281
GI	No	0.275	0.63832	2.78493	1.127
SUB	No	0.193	0.59344	0.6117	-1.362

CLA	No	0.235	0.59344	4.44135	-1.234
CA1	No	0.155	0.58893	0.46173	-1.489
CA2	No	0.304	0.63832	0.60503	-1.06
CA3	No	0.302	0.63832	0.75689	-1.064
DG	No	0.211	0.59344	0.29627	-1.299
FC	No	0.234	0.59344	0.60005	-1.233
LEC	No	0.409	0.71811	0.95904	-0.847
PER35	No	0.072	0.40892	1.98647	-1.964
PER36	No	0.026	0.20745	3.01856	-2.457
NAcc	No	0.608	0.78503	1.00766	-0.524
NAcsh	No	0.966	0.96346	1.72713	-0.043
VSRu	No	0.566	0.76544	0.2234	-0.589
CPu	No	0.918	0.94214	0.55739	0.104
BFRu	No	0.686	0.80494	1.41704	-0.412
Sep	No	0.533	0.76488	1.74176	0.637
GPe	No	0.493	0.76488	0.19538	-0.703
VP	No	0.636	0.78808	0.9676	0.482
Amu	No	0.642	0.78808	0.61528	-0.473
LHb	No	0.238	0.59344	3.20377	-1.226
MHb	No	0.168	0.59178	3.07771	-1.441
RT	No	0.53	0.76488	0.54299	0.642
ZI	No	0.635	0.78808	1.11206	-0.483
ANT	No	0.304	0.63832	3.06348	-1.061
DLG	No	0.61	0.78503	2.25239	-0.521
ILM	No	0.512	0.76488	3.08916	-0.67
LDvl	No	0.126	0.52913	1.8398	-1.608
LP	No	0.039	0.26597	2.78297	-2.258
MD	No	0.105	0.46544	3.53251	-1.714
MG	No	0.02	0.17731	0.82753	-2.715
PoC	No	0.071	0.40892	3.82466	-1.926
VMID	No	0.556	0.76488	1.89594	-0.601
VL	No	0.399	0.71811	2.00026	-0.866
VM	No	0.841	0.9068	1.70747	0.204
VPL	No	0.526	0.76488	2.06687	0.689
VPM	No	0.522	0.76488	2.98725	-0.654
HThu	No	0.584	0.77662	1.56445	-0.558
PAG	No	0.178	0.59178	0.76704	-1.414
PRT	No	0.414	0.71811	1.24801	-0.843
SN	No	0.878	0.93407	0.18994	-0.156
SuD	No	0.349	0.66302	0.58637	-0.971
SuG	No	0.328	0.65	0.53624	-1.016
VTA	No	0.921	0.94214	0.96023	0.101
BSu	No	0.212	0.59344	0.80003	-1.304

Table S4. Statistics of baseline vs. SHBT RNAscope *Oxtr* cell counts t-tests with Two-stage linear step-up procedure of Benjamini, Krieger and Yekutieli method for FDR correction (Q=1%, threshold: No P values marked as "discoveries").

Region	Discovery?	P value	q value	Cohen's d	t value
OB	No	0.283	0.69185	4.10277	-1.115
PIR1	No	0.492	0.69185	1.02034	-0.703
PIR2	No	0.316	0.69185	1.9731	-1.034
PIR3	No	0.405	0.69185	1.62644	-0.854
Au1	No	0.342	0.69185	3.41899	-0.978
Au2d	No	0.327	0.69185	3.55631	-1.008
Au2v	No	0.316	0.69185	4.53322	-1.033
VMID	No	0.4	0.69185	2.13664	-0.864
V1	No	0.038	0.43093	1.6228	-2.361
V2L	No	0.273	0.69185	1.24167	-1.144
S1bf	No	0.565	0.70149	1.85514	-0.587
S1dz	No	0.384	0.69185	0.48975	-0.896
S1f	No	0.544	0.69185	0.60308	-0.619
S1fl	No	0.38	0.69185	0.67717	-0.902
S1hl	No	0.34	0.69185	0.4554	-0.994
S1tr	No	0.548	0.69185	2.87287	-0.613
S2	No	0.46	0.69185	1.44024	0.814
M1	No	0.022	0.43093	0.51566	-2.577
M2	No	0.058	0.43093	1.38471	-2.08
lPPC	No	0.704	0.77922	5.69639	-0.387
mPPC	No	0.689	0.77922	5.38871	-0.408
PtP	No	0.064	0.43093	4.74507	-2.036
TeA	No	0.373	0.69185	2.19806	-0.918
Endo	No	0.261	0.69185	1.53995	-1.164
Cg1	No	0.512	0.69185	0.8748	-0.671
Cg2	No	0.822	0.87392	0.53137	-0.228
MFC	No	0.315	0.69185	1.22273	-1.041
LO	No	0.347	0.69185	1.16981	-0.975
VL	No	0.062	0.43093	1.43635	-2.044
VO	No	0.057	0.43093	1.75851	-2.149
RSD	No	0.699	0.77922	2.22607	-0.393
RSG	No	0.412	0.69185	1.00435	-0.84
AId	No	0.359	0.69185	3.14162	-0.944
AIp	No	0.501	0.69185	1.098	-0.689
AIv	No	0.278	0.69185	1.70346	-1.123
DI	No	0.633	0.74125	1.70307	0.486
GI	No	0.63	0.74125	1.45967	0.518
SUB	No	0.026	0.43093	2.46273	-2.53

CLA	No	0.507	0.69185	0.89213	-0.679
CA1	No	0.231	0.69185	0.84952	-1.241
CA2	No	0.545	0.69185	1.22549	-0.617
CA3	No	0.756	0.82547	0.90928	-0.316
DG	No	0.997	1	0.52732	0.004
FC	No	0.546	0.69185	1.14389	-0.616
LEC	No	0.256	0.69185	1.87581	-1.176
PER35	No	0.213	0.69185	3.39682	-1.293
PER36	No	0.321	0.69185	4.47037	-1.023
NAcc	No	0.529	0.69185	0.69894	0.646
NAcsh	No	0.772	0.8317	1.45182	-0.295
VSRu	No	0.121	0.69185	0.18426	-1.661
CPu	No	0.232	0.69185	0.23629	-1.239
BFRu	No	0.589	0.71032	1.19436	-0.551
Sep	No	0.513	0.69185	1.20935	-0.669
GPe	No	0.019	0.43093	0.09634	2.628
VP	No	0.024	0.43093	1.15226	-2.509
Amu	No	0.041	0.43093	2.13149	-2.234
LHb	No	0.529	0.69185	2.84691	-0.644
RT	No	0.444	0.69185	0.2789	-0.784
ZI	No	0.28	0.69185	0.453	-1.115
ANT	No	0.035	0.43093	4.235	-2.355
DLG	No	0.374	0.69185	1.161	-0.917
ILM	No	0.485	0.69185	2.95843	-0.714
LDvl	No	0.951	0.97267	1.87887	0.062
LP	No	0.537	0.69185	2.61534	-0.631
MD	No	0.898	0.94232	2.87123	-0.131
MG	No	0.309	0.69185	0.10671	-1.055
PoC	No	0.345	0.69185	2.60833	-0.972
VM	No	0.441	0.69185	1.26716	-0.789
V2M	No	0.673	0.77683	4.85204	-0.429
VLO	No	0.944	0.97267	1.30982	-0.072
VPL	No	0.354	0.69185	0.77111	-0.954
VPM	No	0.362	0.69185	1.12866	-0.937
HThu	No	0.158	0.69185	1.80812	-1.477
PAG	No	0.045	0.43093	1.04448	-2.192
PRT	No	0.372	0.69185	0.34899	-0.921
SN	No	0.483	0.69185	0.10954	0.793
SuD	No	0.52	0.69185	0.19643	0.724
SuG	No	0.573	0.70149	0.11129	-0.578
VTA	No	0.463	0.69185	0.82685	-0.753
BSu	No	0.265	0.69185	0.92102	-1.157
